# Integrated analysis of direct and proxy genome wide association studies highlights polygenicity of Alzheimer’s disease outside of the APOE region

**DOI:** 10.1371/journal.pgen.1010208

**Published:** 2022-06-03

**Authors:** Javier de la Fuente, Andrew D. Grotzinger, Riccardo E. Marioni, Michel G. Nivard, Elliot M. Tucker-Drob

**Affiliations:** 1 Department of Psychology, University of Texas at Austin, Texas, United States of America; 2 Population Research Center and Center on Aging and Population Sciences, University of Texas at Austin, Texas, United States of America; 3 Psychiatric and Neurodevelopmental Genetics Unit (PNGU) and the Center for Genomic Medicine, Massachusetts General Hospital, Boston, Massachusetts, United States of America; 4 Centre for Genomic and Experimental Medicine, Institute of Genetics and Cancer, University of Edinburgh, United Kingdom; 5 Department of Biological Psychology, VU University Amsterdam, the Netherlands; Emory University, UNITED STATES

## Abstract

Recent meta-analyses combining direct genome-wide association studies (GWAS) with those of family history (GWAX) have indicated very low SNP heritability of Alzheimer’s disease (AD). These low estimates may call into question the prospects of continued progress in genetic discovery for AD within the spectrum of common variants. We highlight dramatic downward biases in previous methods, and we validate a novel method for the estimation of SNP heritability via integration of GWAS and GWAX summary data. We apply our method to investigate the genetic architecture of AD using GWAX from UK Biobank and direct case-control GWAS from the International Genomics of Alzheimer’s Project (IGAP). We estimate the liability scale common variant SNP heritability of Clinical AD outside of *APOE* region at ~7–11%, and we project the corresponding estimate for AD pathology to be up to approximately 23%. We estimate that nearly 90% of common variant SNP heritability of Clinical AD exists outside the APOE region. Rare variants not tagged in standard GWAS may account for additional variance. Our results indicate that, while GWAX for AD in UK Biobank may result in greater attenuation of genetic effects beyond that conventionally assumed, it does not introduce appreciable contamination of signal by genetically distinct traits relative to direct case-control GWAS in IGAP. Genetic risk for AD represents a strong effect of *APOE* superimposed upon a highly polygenic background.

## Author summary

In this article we demonstrate that common approaches for combining direct and proxy GWAS data produce dramatic underestimates of heritability, and we introduce a novel multivariate method for recovering unbiased estimates. Applying our method to direct and proxy GWAS data for Alzheimer’s disease we obtain estimates of liability-scale SNP heritability of AD outside of APOE region that are more than double the most recent estimates. Our analysis of local SNP heritability indicates that nearly 90% of common variant risk for Clinical AD represent a polygenic signal that is relatively diffusely distributed across the genome outside of the APOE region. We add substantial clarity to the scientific understanding the genetic architecture of AD, by introducing and validating new methodology that can be widely applied by researchers seeking to incorporate family information into GWAS.

## Introduction

Genome-wide association studies (GWAS) of proxy-phenotypes using family history of disease (GWAX) can substantially boost power when combined with traditional case-control GWAS across a range of disease traits [1,2]. The benefits of GWAX for enhancing GWAS discovery have been particularly pronounced in the context of late-onset Alzheimer’s Disease (AD) [3–5], a neurocognitive disorder of aging that is clinically characterized by significant cognitive declines that interfere with independence in everyday activities, and biologically characterized by amyloid-predominant neuritic plaques, tau-predominant neurofibrillary tangles, and neurodegeneration [6–10]. Recent meta-analyses combining GWAX with direct GWAS have expanded the number of AD-relevant loci far beyond the well-established *APOE* variant, to 75 loci in total [5]. However, despite a twin-based heritability estimates of approximately 60% [11], these studies indicate very low common variant SNP heritability after excluding the *APOE* region, with the most recently reported estimate of 2.5% coming from the largest combined GWAX-GWAS meta-analysis of AD to date [4]. This estimate is noticeably lower than estimates obtained from the application of both LDSC and raw data-based methods in earlier studies that have only included direct case-control designs, albeit in smaller samples [12–14]. If valid, this low SNP heritability estimate may call into question the prospects of continued progress in genetic discovery for AD within the spectrum of common variants.

Here, we revisit the genome-wide architecture of AD. In scrutinizing recent approaches for estimating SNP heritability from GWAX, we find that even under scenarios in which the assumptions of the standard GWAX model are met, commonly used approaches for combining GWAX and GWAS data produce dramatic underestimates of SNP heritability. We develop a flexible data-driven multivariate framework for the accurate estimation of SNP heritability from GWAS and GWAX summary data, even in the presence violations of the standard GWAX assumptions. Using GWAS data from the International Genomics of Alzheimer’s Project (IGAP) [12] and GWAX data from UK Biobank [3] our multivariate method yields substantially increased heritability estimates for clinically defined AD relative to the recently reported estimates from naïve meta-analysis of GWAS and GWAX data. We find that common genetic variants distributed outside of the *APOE* region account for between approximately 7% and 11% of variation in liability for Clinical AD. Informed by prevalence rates from epidemiological and molecular imaging data we project that common variants distributed outside of the APOE region may account for approximately 23% of variation in liability for biological AD (Alpha+ and Tau+). Our cumulative analysis of local SNP heritability of AD indicates a relatively continuous increase in genetic signal across the genome with a sharp discontinuity at the APOE locus, indicating that while the *APOE* region accounts for approximately 10% of common variant effects on AD, the remaining common variant risk for AD represents polygenic signal that is diffusely distributed across the genome.

## Results

### Overview of methods

Recent large scale meta-analyses combining direct-GWAS and GWAX data have used either inverse variance weighted meta-analysis of regression coefficients [3,15] or sample size weighted meta-analysis of Z statistics [4,16]. For the inverse variance approach, the standard correction factor for GWAX of the phenotype of a single first degree relative using the offspring genotype is to multiply regression coefficients and their SEs by 2.0 to correct for 50% attenuation due to 50% genetically relatedness (note that in the R^2^ metric, this amounts to 75% attenuation). A similar correction to the Z statistics approach is possible (section 3 in S1 Supplementary Note) but not typically made. However, even with such corrections, estimation of SNP heritability from the summary statistics produced by either method produces estimates that are severely biased, unless the sample size input is further corrected (section 4 in S1 Supplementary Note). Moreover, GWAX estimates will be attenuated by more than the 50% assumed by the standard correction under a wide range of circumstances such as those in which: a proportion of genotyped individuals report on the phenotypes of their step or adoptive parents (such that average genetic relatedness of phenotyped and genotyped individuals falls below 50%); individuals are not well-informed about, misremember or confuse their parents’ phenotype or disease status (such that heritability of the GWAX phenotype is attenuated, or contaminated by other heritable phenotypes); or the average diagnostic quality or criteria differ between proxy reports of historical disease status and direct GWAS of carefully screened case-control sample (such that heritability of the GWAX phenotype is attenuated). Here, we develop a multivariate model that directly estimates the appropriate correction empirically from the GWAX and direct GWAS summary data in order to produce unbiased estimates of SNP heritability and SNP effects without manual correction of effect size estimates, standard errors, or sample sizes.

Our multivariate model (section 5 in S1 Supplementary Note and S1 Fig) integrates summary data from three sources: direct GWAS, maternal GWAX, and paternal GWAX. In the model, the total genetic propensity toward AD risk is represented as a latent factor, *F*, that is specified to affect the direct GWAS phenotype and two GWAX phenotypes according to the following system of regression equations

$$\left[\begin{array}{c} Y_{direct}\\ Y_{mat}\\ Y_{pat} \end{array}\right]=\left[\begin{array}{c} \lambda_{direct}\\ \lambda_{mat}\\ \lambda_{pat} \end{array}\right]F+\left[\begin{array}{c} u_{direct}\\ u_{mat}\\ u_{pat} \end{array}\right],$$

where the *λ* coefficients relate *F* to measured phenotypes *y*, and the *u* terms are residual genetic propensities toward each of the measured phenotypes that are independent of *F*, and uncorrelated with one another and with *F*. We specify the model with the minimal identification constraint that *λ*_*direct*_ = 1 such that *F* takes on the scale of the direct GWAS phenotype, and $\sigma_{F}^{2}$ can be interpreted as an unbiased estimate of the SNP heritability of the meta-analyzed phenotype in the direct GWAS metric. Note that the standard GWAX approach[1] implicitly treats *λ*_*mat*_ = *λ*_*pat*_ = .5 and $\sigma_{u_{Direct}}^{2}=\sigma_{u_{Mat}}^{2}$ = and $\sigma_{u_{Pat}}^{2}$ = 0. Thus, under our more flexible multivariate parameterization in which these terms are freely estimated, the departure of *λ*_*mat*_ and *λ*_*pat*_ from .5 and departure of the $\sigma_{u}^{2}$ terms from 0 indicate departure of the empirical data from the standard GWAX assumptions. If we are willing to assume that the direct GWAS represents the pure signal of interest, uncontaminated from other heritable traits (e.g. other forms of dimension), we can fix $\sigma_{u_{Direct}}^{2}$ to 0, in which case the multivariate model becomes similar to the MTAG model [17,18].

To estimate meta-analytic summary statistics using this multivariate model (section 6 in S1 Supplementary Note), we expand it to include the effect of an individual genetic variant, *x*, on the *F* as follows

F=γx+e,

where *γ* is an unstandardized regression coefficient and *e* is a residual. As described in section 7 of in S1 Supplementary Note, a SNP-specific heterogeneity statistic (Q_SNP_) is computed to gauge the extent to which the regression coefficient *γ* does not well account for the pattern of associations between that SNP and the individual GWAS and GWAX.

Models are estimated in Genomic SEM [18] using a two-stage approach. In the first stage, the empirical liability-scale genetic covariance matrix and its sampling covariance matrix are estimated. In the second stage, the model is fit to the matrices using the diagonally weighted least squares (WLS) fit function with sandwich correction, as described in Grotzinger et al. [18]. Beyond its flexible capabilities for user-specified modeling, Genomic SEM is advantageous for its accommodation of unknown and varying degrees of sample overlap. This may be particularly advantageous for instances in which individuals included in the GWAX are related to those in the direct GWAS, or when users seek to incorporate GWAX from relatives who may themselves be related (e.g. both brothers and sisters of the directly genotyped individuals [18]). We refer to our multivariate model as a Genomic SEM Relaxed Model, in that it uses Genomic SEM to relax the conventional assumption that *λ*_*mat*_ = *λ*_*pat*_ = .5. A complete tutorial for our method, including detailed code, can be found in the Tutorials tab of https://github.com/GenomicSEM/GenomicSEM/wiki.

### Simulation results

We simulated genome-wide summary statistics for direct GWAS and maternal and paternal GWAX manipulating the attenuation coefficients (λ) for maternal and paternal GWAX assuming either continuous or binary traits. In experimental condition 1, the simple GWAX model assumptions hold (i.e., λ_mat_ = λ_pat_ = .5). In experimental condition 2, we consider a strong deviation from the simple GWAX model, (i.e., λ_mat_ = λ_pat_ = .25). In experimental condition 3 we set the attenuation coefficients to mimic those empirically estimated from our multivariate model using real GWAS and GWAX data (i.e., λ_mat_ = .463, λ_pat_ = .366). Although our simulations refer to maternal and paternal GWAX, it is of note that our multivariate model can incorporate family history GWAX data across diverse degrees of genetic relatedness. We compared the Genomic SEM approach to a conventional uncorrected approach in which GWAS and GWAX summary data are meta-analyzed without explicit consideration of whether they were derived from direct or proxy data sources (cf. by Jansen et al. [16] and Wightman et al. [4]) and the conventional corrected approach (reflective of those taken by Marioni et al. [3], and Bellenguez et al. [5].

**Fig 1:**
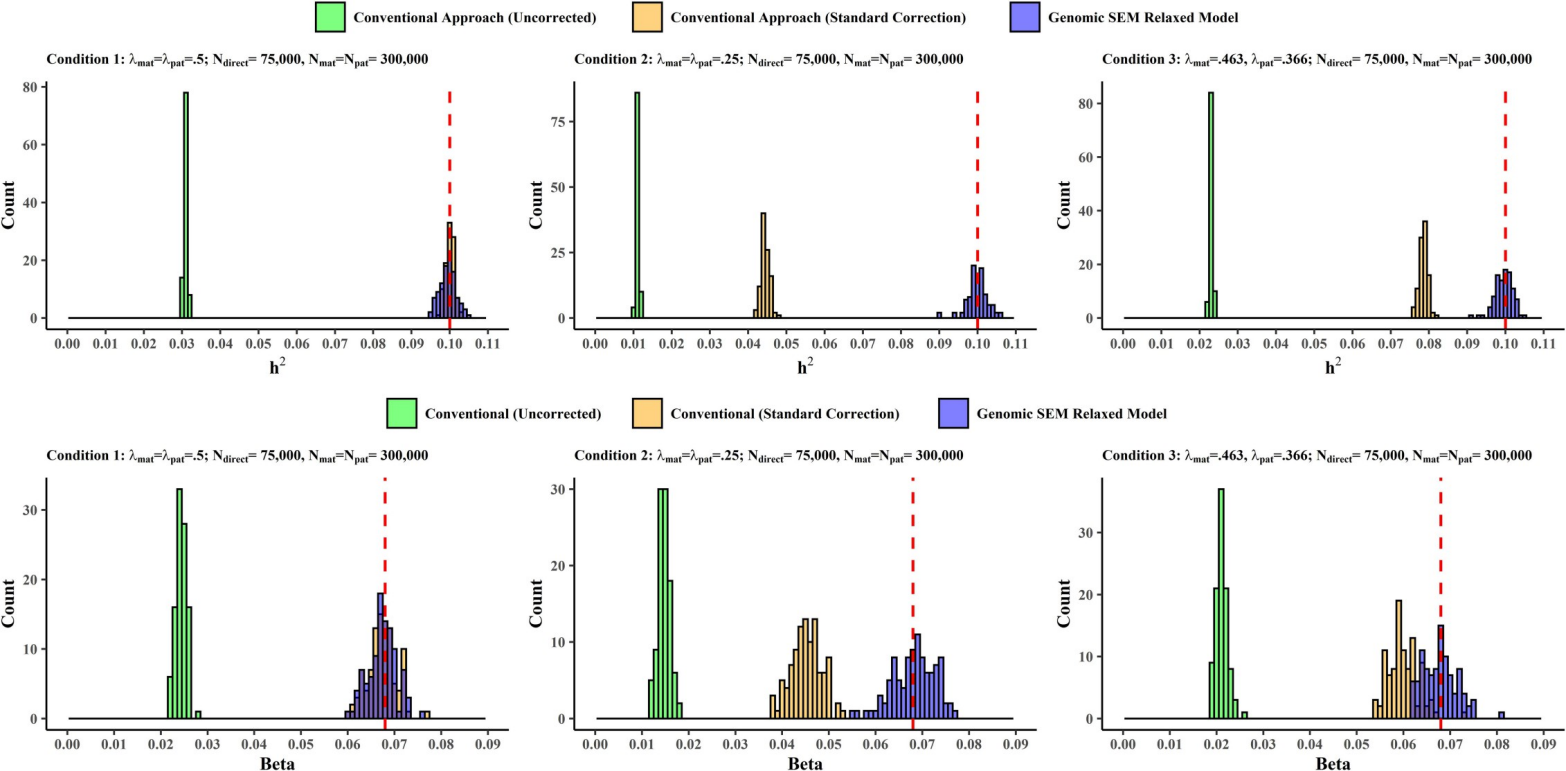
Simulation Results for continuous phenotypes. Distribution of SNP heritability estimates (top row) and individual SNP effects (bottom row) for Conventional (Uncorrected), Conventional (Standard Correction), and Genomic SEM Relaxed Model across conditions. The vertical dashed red lines indicate the true parameter value in the population. Complete simulation results for all conditions for continuous traits are reported in S2, S3, S10 and S11 Tables.

**Fig 2:**
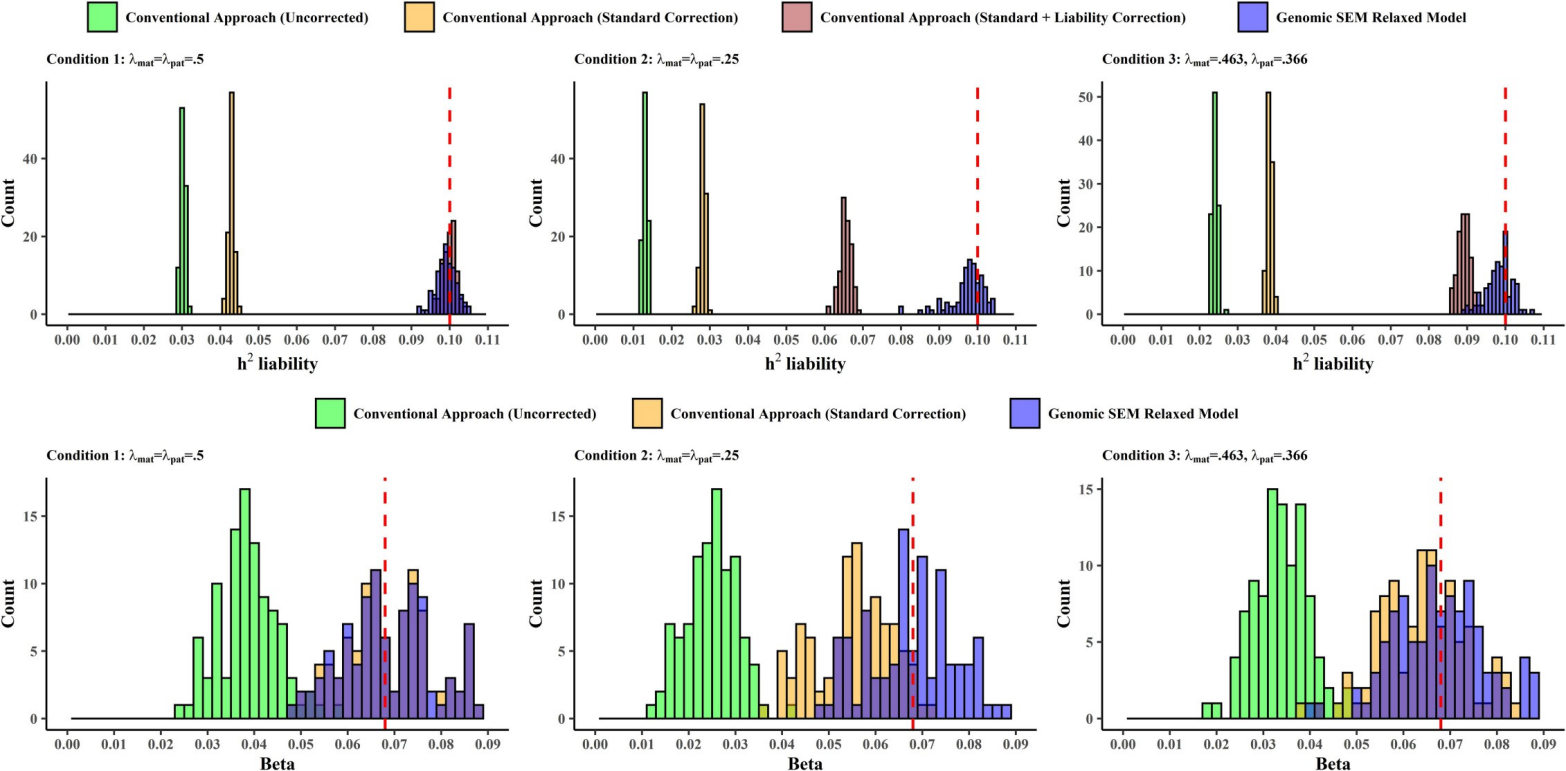
Simulation Results for binary phenotypes. Distribution of liability-scale SNP heritability estimates (top row) and individual SNP effects (bottom row) for Conventional (Uncorrected), Conventional (Standard Correction), Conventional (Standard + Liability Correction), and Genomic SEM Relaxed Model across conditions. The vertical dashed red lines indicate the true parameter value in the population. Complete simulation results for all conditions are reported in S2 and S3 Tables.

The top row of Figs 1 and 2 presents simulation results for continuous phenotypes (Fig 1) and binary phenotypes (Fig 2) with respect to heritability estimates from conditions in which sample size is held constant and the λ coefficients vary, reflecting different degrees of deviation from the simple GWAX assumption that λ_mat_ = λ_pat_ = .5. Note that liability scale heritability estimates are reported for binary phenotypes. Supplemental results for the recovery of SNP heritability for continuous traits in conditions with varying sample size are reported in S11 Table and displayed in S2 Fig. The multivariate Genomic SEM approach provided essentially unbiased heritability estimates ($h_{F}^{2}$) across all conditions, outperforming the conventional (uncorrected) approach in all conditions, and outperforming the conventional (uncorrected) approach, the conventional approach with standard correction, and the conventional approach with standard and liability correction in conditions in which the assumptions of the simple GWAX model did not hold, for both continuous (Percent Bias Error, %BE, range for conventional [uncorrected] approach: 76.97%-88.91%; %BE range for conventional [standard correction]: 21.39%-55.56%; %BE range Genomic SEM relaxed model: 0.04%-0.36%), and binary traits (%BE, range for conventional [uncorrected] approach: 75.93%-86.94%; %BE range for conventional [standard correction]: 61.68%-71.85%; %BE range for conventional [standard + liability correction]: 10.82%-34.48%; %BE range Genomic SEM relaxed model: 1.69%-2.65%). As expected, the conventional approach with the standard correction (for continuous traits), and the conventional approach with standard and liability corrections (for binary traits), performed similarly to the Genomic SEM approach when the standard assumption that λ_mat_ = λ_pat_ = .5 held. Bias in the conventional approaches was related to the extent to which the population values of λ_mat_ and λ_pat_ deviated from .5.

The bottom row of Figs 1 (continuous traits) and 2 (binary traits) presents simulation results with respect to individual SNP effects for conditions in which sample size is held constant and the λ coefficients vary. Supplemental results for recovery of individual SNP effects for continuous traits with varying sample size across conditions are provided in S12 Table and S3 Fig. Results for the recovery of individual SNP effects were consistent with those for the recovery of heritability. Our multivariate Genomic SEM approach exhibited consistently unbiased performance across all conditions (%BE range 0.32%-1.49%), outperforming the conventional uncorrected and corrected approaches in all conditions for continuous traits (Percent Bias Error, %BE, range for conventional [uncorrected] approach: 64.19%-78.33%; %BE range for conventional [standard correction]: 1.01%-33.46%; %BE range Genomic SEM relaxed model: 0.23%-0.87%), and the conventional uncorrected and corrected approach in conditions in which the assumptions of the simple GWAX model did not hold for binary traits (Percent Bias Error, %BE, range for conventional [uncorrected] approach: 50.40%-63.44%; %BE range for conventional [standard correction]: 5.88%-18.75%; %BE range Genomic SEM relaxed model: 0.29%-0.59%). When the simple GWAX model assumptions held, the Genomic SEM approach and the conventional approach with the standard correction were both unbiased, but the conventional approach without correction remained biased. Bias in the conventional approaches was related to the extent to which the population values of λ_mat_ and λ_pat_ deviated from .5.

We additionally simulated GWAS and GWAX summary statistics under a null scenario in which genetic covariances among GWAS and GWAX were set to 0, which we analyzed using the multivariate Genomic SEM model. In this scenario, the GWAS and GWAX represent genetically distinct phenotypes, such that a factor representing their shared genetic architecture is expected to produce a heritability estimate of 0. As expected, the mean SNP heritability across replications was 0%, indicating an unbiased parameter estimate. Moreover, SNP heritability was significant at *p* < .05 in none of hundred replications (S9 Table), compared to an expected frequency of 5, indicating appropriate, if not conservative, Type-I error control.

### Multivariate model of direct GWAS and GWAX of Alzheimer’s disease

We applied our multivariate model to empirical summary data from the direct case-control GWAS of AD in IGAP [12] and GWAX of maternal and paternal AD in UK Biobank [3]. Key descriptive statistics for these three contributing datasets are reported in the top portion of Table 1, and multi-trait LDSC results (cross trait intercepts and genetic correlations) are provided in S4 Fig. Note that all LDSC estimates reported in Table 1 and S4 Fig are derived from LD scores based on common variants (MAF ≥ .05) outside of the MHC and *APOE* regions, using the AD population prevalence rate of 5%. Cross-trait intercepts among IGAP and the maternal and paternal GWAX were all ~0, indicating that there was effectively no sample overlap or dependency between LDSC estimates. Genetic correlations were close to 1.0 between IGAP and maternal AD (rG = 0.915, SE = 0.153, *p* < 0.001) and paternal AD (rG = 0.842, SE = 0.206, *p* < 0.001) in UKB. The genetic correlation between maternal and paternal AD was also very high (rG = 0.815, SE = 0.253, *p* = 0.001).

**Table 1:** Descriptive statistics for contributing cohorts and previous meta-analyses of case-control and proxy-phenotypes of Alzheimer’s disease and LDSC output.

				LDSC^a^										
	Phenotype	Mean χ^2^	λ_GC_	h^2^_liability_ (prevalence = 5%)	SE	Z	Intercept	Maternal Cases	Maternal Controls	Paternal Cases	Paternal Controls	Direct Cases	Direct Controls	N reported
Contributing Summary Data	IGAP—Stage I	1.117	1.089	0.073	0.012	6.083	1.025	-	-	-	-	21,982	41,944	63,926
	UKB Maternal	1.049	1.032	0.017	0.004	4.250	1.006	27,696	260,980	-	-	-	-	288,676
	UKB Paternal	1.031	1.029	0.012	0.006	2.000	1.009	-	-	14,338	245,941	-	-	260,279
Meta-analyses	Multivariate Model	1.139	1.079	0.069	0.008	8.625	1.001	27,696	260,980	14,338	245,941	21,982	41,944	-
	Marioni et al. (2019)	1.142	1.085	0.023	0.003	7.333	1.025	27,696	260,980	14,338	245,941	25,580	48,466	388,324
	Jansen et al. (2019)	1.114	1.068	0.020	0.002	9.500	1.000	47,793^b^	328,320^b^	-	-	24,087	55,058	455,258

^a^LDSC-derived results are restricted to HapMap3 SNPs only, excluding the MHC and *APOE* (chr:19, bp 45,116,911: 46,318,605) regions. LDSC-derived results are derived from for the multivariate model are obtained by submitting the summary statistics from our multivariate meta-analysis to LDSC.

^b^These Ns refer to the total numbers of proxy cases and proxy controls (i.e., maternal + paternal cases and maternal + paternal controls).

*Note*: Heritability estimates reported here are on a liability scale, based on a population prevalence of 5%. Elsewhere in this paper, we consider sensitivity of liability scale heritability estimates to other values, and to the possibility of undiagnosed AD within control participants, and we specifically compare estimates to those obtained with a population prevalence of 5% used by Wightman et al. [4] to produce their estimate of h^2^_liability_ of 2.5%. Heritability presented for GWAX phenotypes and Marioni and Jansen meta-analyses are naïve, and have not been corrected for the indirect nature of the GWAX.

**Fig 3:**
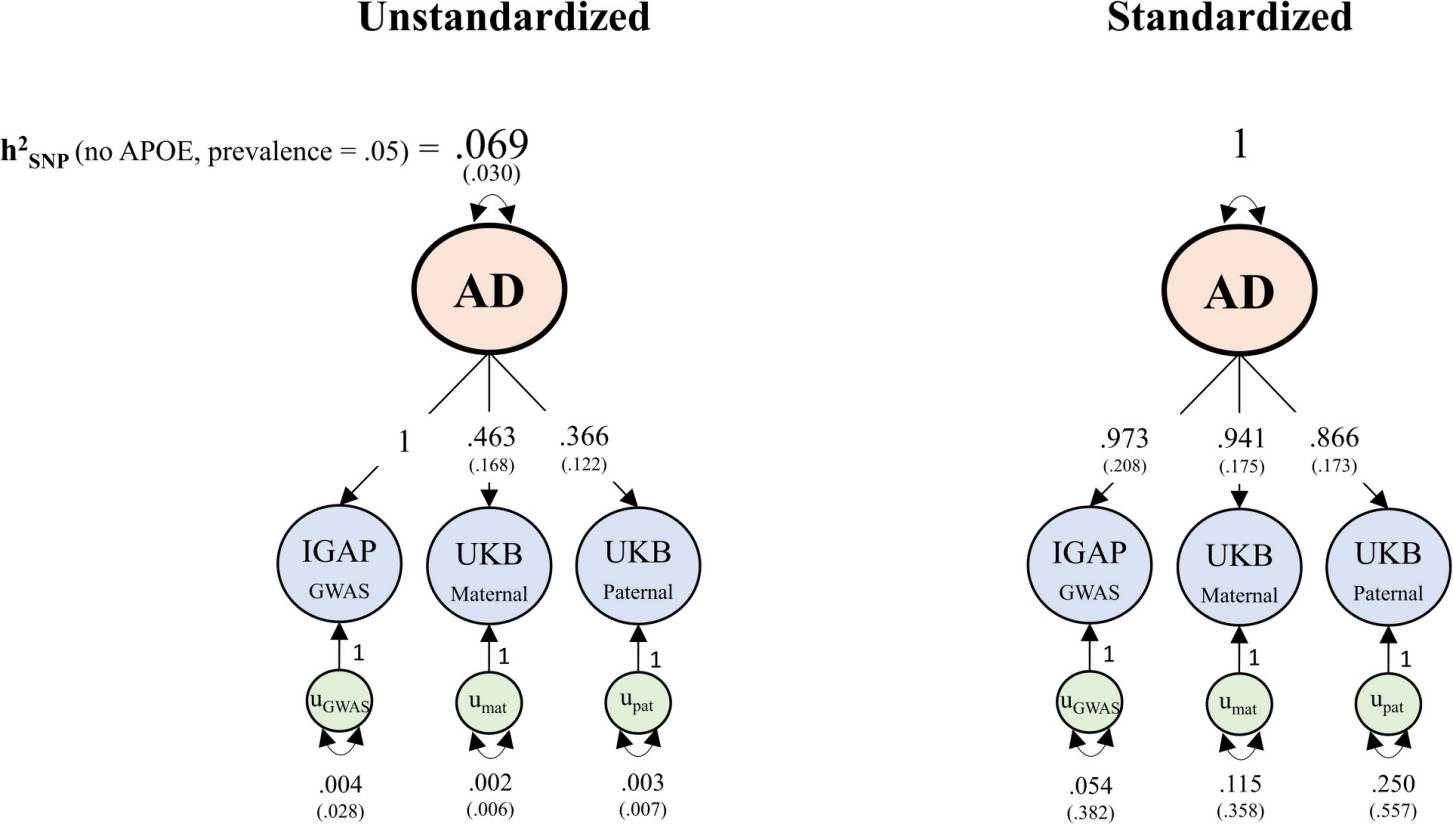
Unstandardized (left) and standardized (right) empirical results from multivariate genetic analysis of Alzheimer’s disease. The liability-scale SNP heritability estimate of 6.9% is on the scale of the direct GWAS, and is for common variants (MAF ≥ .01) outside of the MHC and *APOE* regions, using the AD population prevalence of 5%. *u* = residual genetic variance.

In the bottom portion of Table 1 we provide key descriptive of summary statistics from our multivariate meta-analysis and from two recent meta-analyses that have combined GWAS and GWAX AD data [3,16]. Estimates from the full multivariate model of AD are displayed in Fig 3. In the model itself, the liability-scale SNP heritability estimate was 6.95% (SE = 3%) assuming a population prevalence of 5%, over double that estimated by application of LDSC to the summary statistics from the other two recent AD meta-analyses, using their reported sample sizes, assuming the same population prevalence. A version of the model that constrained residual variance of the direct GWAS to 0 (reflecting the assumption that the direct GWAS does not contain ancillary genetic signal unrelated to AD) did not produce a significant decrement in model fit (χ^2^(1) = .021, *p* = .89) and produced a slightly larger liability scale SNP heritability estimate (7.17%) with a substantially smaller standard error (SE = 1.21%).

Applying LDSC to GWAS summary statistics produced by our multivariate method produced a very similar liability-scale SNP heritability estimate (6.9%, SE = .8%; Table 1; assuming a 5% population prevalence rate) to that produced by the model itself. In the full model, the unstandardized loading for maternal AD (*λ*_*mat*_ = .463) was slightly attenuated with respect to the expectation under the standard GWAX model (*λ*_*mat*_ = .5). The unstandardized loading for paternal AD (*λ*_*pat*_ = .366) reflected approximately 27% attenuation of regression effects (i.e.[.5-.366]/.5) and approximately 46.42% attenuation of R^2^ and liability-scale h^2^ estimates ([.5^2^-.366^2^]/[.5^2^]) relative to the expectation under the standard model (*λ*_*pat*_ = .5). Allowing the *λ*_*mat*_ and *λ*_*pat*_ parameters to be freely estimated can avoid the potential for bias stemming from violations of the standard assumption, in this case particularly for paternal effects, when estimating genome wide meta-analytic summary statistics. Residual variances for the direct GWAS and both GWAX were trivial, indicating that the GWAS and GWAX were not tapping genetically distinct phenotypes to an appreciable extent. We note that the liability-scale heritability estimates reported in this section are based on an assumed AD population prevalence rate of 5%, as per the LDSC analyses by Wightman et al. [4]. Next, we consider sensitivity of the heritability estimate to different assumptions regarding the sample and population prevalence rates of AD.

### Liability scale heritability across a range of assumed population prevalence rates

Heritability estimates for case-control traits, such as AD, are based on a liability threshold model, which assumes a continuously distributed liability toward the binary phenotype in the population. Estimates of liability-scale SNP heritability are sensitive to assumptions about the lifetime prevalence of the disorder in the population and the extent to which unaffected individuals have been successfully screened for the disorder (see Methods). When population prevalence rates are high or when control participants are unscreened, differences in allele frequencies between cases and controls represent less extreme comparisons along regions of the liability distribution. In such circumstances, the inferred liability scale heritability is higher than would be inferred from the same case-control difference in allele frequencies for a disorder with a lower population prevalence or when control participants have been carefully screened. This issue is particularly germane to the study of AD, a disorder whose: (i) clinical prevalence rate increases from less than 1% in middle adulthood to approximately 30% by old age [19], (ii) that is known to go undetected at high rates for decades prior to diagnosis due to ancillary factors (e.g. educational attainment) unrelated to biological severity [20], and (iii) whose pathophysiological basis may be more than twice as prevalent as its clinical diagnosis at any given age [10].

**Fig 4:**
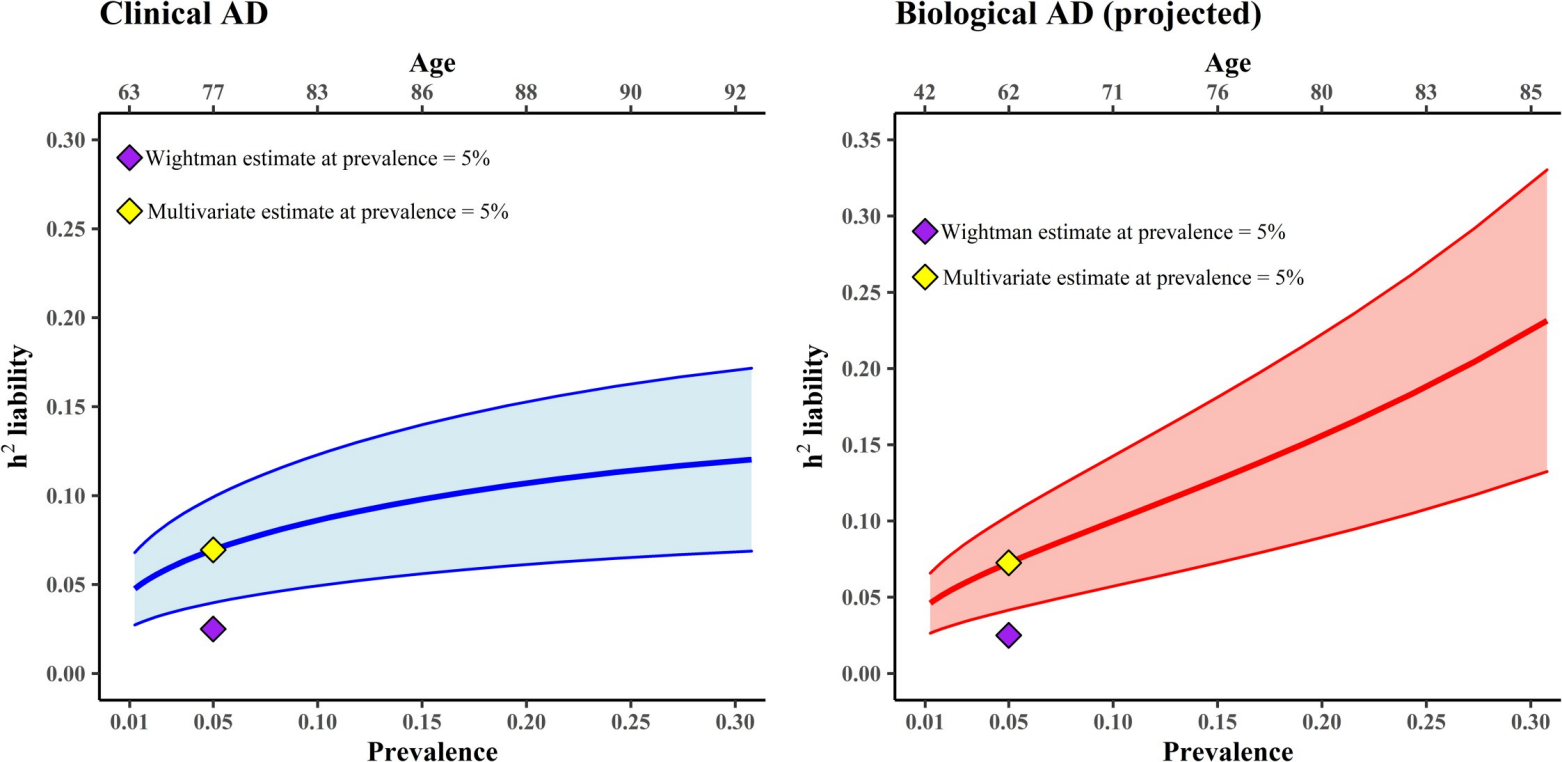
Estimated common variant liability-scale SNP heritability of AD (outside of the MHC and *APOE* regions) according to different assumptions regarding the population prevalence of Clinical AD and Biological AD (Alpha+ and Tau+). We provide rough age equivalences for each prevalence rate on the top x axis. The purple diamond represents the estimate of 2.5% by Wightman et al. [4], which was based on an assumed population prevalence rate of 5%. The yellow diamond represents the estimate from the multivariate model introduced here, using the same assumed population prevalence rate of 5% (Clinical AD h^2^ liability = 0.069; Biological AD h^2^ liability = 0.073). The shaded area around the line reflects +/- 1 SE of the h^2^ estimate. The steeper shift in the SNP heritability of AD for biological AD compared to clinical AD as a function of population prevalence stems from the correction for undetected biological AD within control participants who primarily only been screened for clinical AD. As the assumed prevalence rate of biological AD increases, the extent of case contamination in control participants increases, and the correction for undetected AD in control participants produces more dramatic increases in the projected heritability. Biological AD prevalence rates are from recently published positron emission tomography data [10].

To gauge the effects of assumptions regarding population prevalence on our liability-scale SNP heritability estimate for AD, we varied the assumed population prevalence rate. We provide rough age equivalents of these prevalence rates based on published epidemiological data for Clinical AD [19], assuming that control participants were appropriately screened. We refer to this estimate as the estimate of heritability of Clinical AD, in that this estimate does not account for biological AD that is likely to exist in a potentially sizable subset of control participants. Estimates from the full multivariate model are displayed in the left panel of Fig 4 (estimates from the application of LDSC to the summary statistics from our multivariate meta-analysis were very similar; S7 Fig). Heritability estimates of Clinical AD increase as the population prevalence rate increases, reaching approximately a liability-scale h^2^ = 12% at prevalence = 30%. We note that Wightman et al. [4], estimate a liability-scale heritability of 2.5% assuming a population prevalence rate of 5%. At this same prevalence rate, the estimate from our full multivariate model is over double (liability h^2^ = 6.95%) that of Wightman et al. This is likely a reflection of the fact that Wightman et al. do not appear to make any correction to their LDSC estimate to account for any attenuation in the GWAX estimates due to their indirect nature.

We also estimated liability-scale SNP heritability (for common variants outside of the MHC and *APOE* regions) across a range of different assumed prevalence rates of biological AD, employing a correction for case contamination (see Methods) in control participants (who were primarily screened for Clinical AD but not AD pathology) and provide rough age equivalents of these prevalence rates for biological AD (Alpha+ and Tau+) from recently published positron emission tomography data [10]. We refer to this estimate as the projected estimate of biological AD heritability. Results are displayed in the right panel of Fig 4. Heritability estimates increase steeply as the prevalence rate increases, reaching approximately a liability-scale h^2^ = 23% at a population prevalence of 30%. The steeper shift in the SNP heritability of AD for biological AD compared to clinical AD stems from our correction for undetected biological AD within control participants who have primarily only been screened for clinical AD. As the assumed prevalence rate of biological AD increases, the extent of case contamination in control participants increases, and the correction for undetected AD in control participants produces more dramatic increases in the projected heritability. The validity of this inference relies on the assumptions that individuals with clinical AD diagnoses are representative of the larger set of individuals with biological AD, and do not constitute a subgroup of those with more severe biological AD. This assumption is supported by a large body of work indicating that ancillary factors, such as educational attainment, are associated with clinical AD rates among individuals with equivalent levels of brain pathology [20].

### Liability scale heritability using stratified LDSC

To allow for potentially uneven contributions of SNPs to heritability across biologically, evolutionarily, and MAF defined categories, we fit our Genomic SEM relaxed model to a genetic covariance matrix derived using stratified LDSC [21] based on 97 baseline annotations [22], assuming 5% population prevalence. We obtained larger, albeit less precise, estimates of liability-scale SNP heritability (h^2^ = 8.94%, SE = 5.17%) than those obtained when using the standard LDSC model (h^2^ = 6.95%, SE = 3%).

### Inferring common variant polygenicity from local SNP heritability

**Fig 5:**
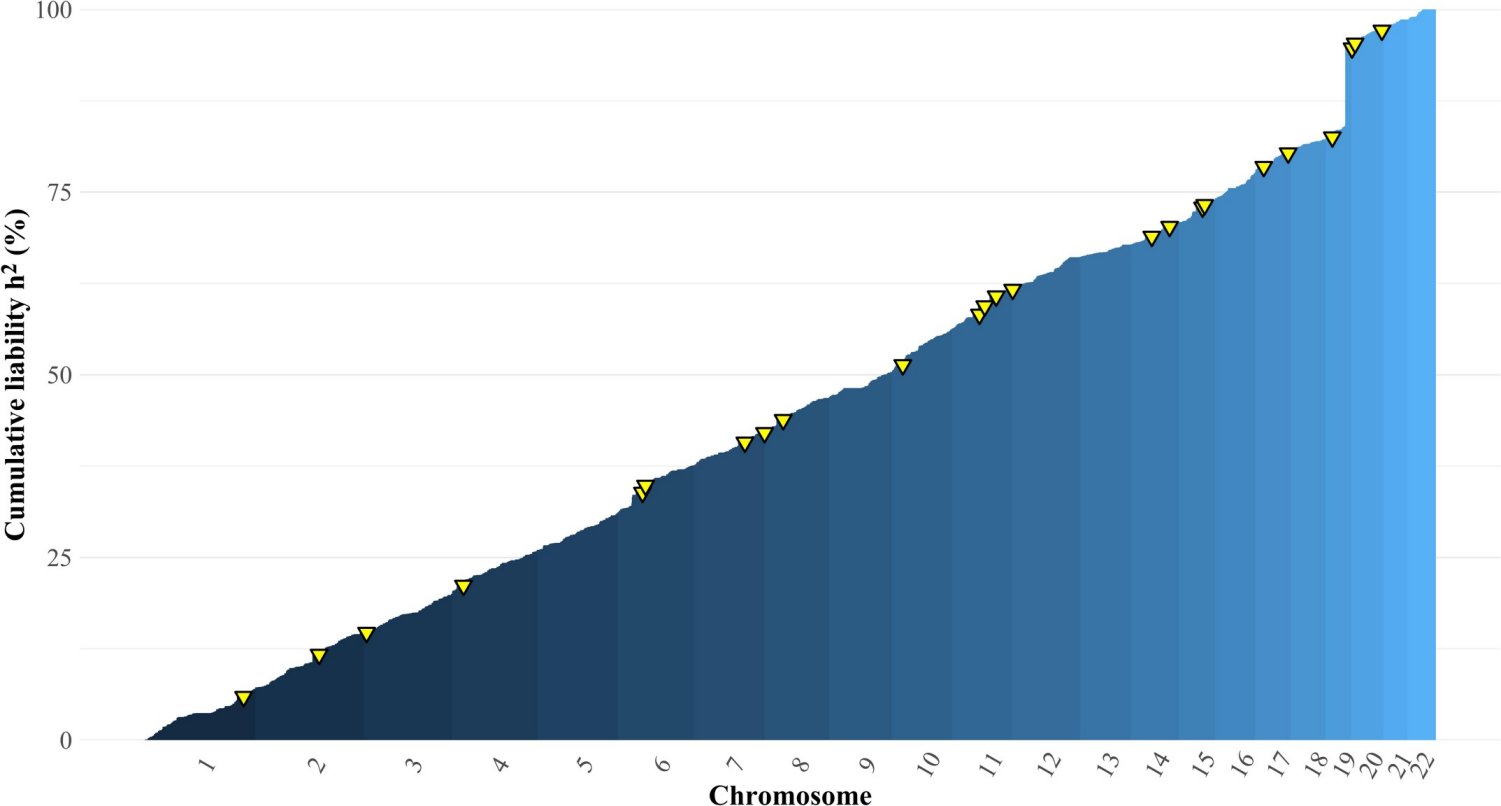
Proportion of cumulative heritability of Clinical AD across the genome, as estimated with HESS (24). Yellow triangles represent genome-wide significant loci from the multivariate GWAS of AD.

Our results indicated relatively high common variant SNP heritability of AD outside of the *APOE* locus. To investigate whether this SNP heritability was attributable to a small number of genomic regions, or distributed more evenly across the genome, we used Heritability Estimation from Summary Statistics (HESS) [23] to estimate local common variant SNP heritability in 1,703 approximately independent blocks across the genome using the summary statistics from our multivariate meta-analysis. Following Sinnott-Armstrong et al. [24], we plot the proportion cumulative heritability of AD liability across the genome in Fig 5. Because HESS allows for negative estimates of local SNP heritability, estimation error is not expected to produce increases in cumulative heritability; only true polygenic signal will produce such increases. It can be seen that heritability accumulates relatively continuously across the entirety of the genome, with a pronounced discontinuity at the *APOE* locus on chromosome 19. This locus accounts for 10.53% of total common variant SNP heritability of AD in the liability scale, indicating that most of common variant SNP heritability is attributable to genetic signal independent of *APOE*. Local SNP heritability in the other loci that were genome-wide significant in the multivariate GWAS was strongly correlated with their GWAS effect sizes (S8 Fig) and accounted for an additional 9.46% of the total common variant liability-scale SNP heritability, leaving 79.98% of the total common variant liability-scale SNP heritability unexplained by genome-wide significant loci. The observations that heritability accumulates relatively continuously outside of the *APOE* locus and that substantial proportion of SNP heritability remains outside of genome-wide significant loci suggests that genetic risk for AD may be affected by core pathways superimposed on a more diffuse polygenic background. Using the conservative estimate of population prevalence of 5% the total heritability of AD on the liability scale as estimated with HESS was 11.09% (9.92% excluding the *APOE* locus). Similar patterns were observed when HESS analyses were limited to GWAS data from IGAP only (S9 Fig).

### Multivariate results for individual SNP effects

We estimated meta-analytic summary statistics containing individual SNP effects on AD in our multivariate model using Genomic SEM, a Manhattan plot for which is provided in S5 Fig, and qqplots for which are provided in S6 Fig. The mean χ^2^ for the common factor GWAS output was 1.139. The mean χ^2^(1) for Q_SNP_ was 1.008, and there were no genome-wide significant (*p* < 5 × 10^−8^) hits for Q_SNP_, indicating little evidence for genome-wide heterogeneity in SNP effects across direct GWAS, maternal GWAX, and paternal GWAX after the empirically derived attenuation coefficients (λ) are taken into account. The LDSC intercepts were all very close to 1.0, indicating that inflation of test statistics was predominately attributable to true polygenic signal rather than population stratification (Table 1).

We identified 282 independent significant SNPs and 93 lead SNPs in a total of 24 genome-wide significant loci associated with AD (S4–S8 Tables). All of these significant loci were previously reported in published meta-analysis of GWAX and direct GWAS of AD by Marioni et al. [3], Jansen et al. [16], or Schwartzentruber et al. [25]. For the genome-wide significant loci, we computed meta-analytic estimates using the inverse variance weighted approach and the Z approach. We applied each approach both naively (i.e. without correction) and with a correction for attenuation due to the indirect nature of the GWAX with the standard correction (S6 Table). As expected, based on the fact that the empirically derived λ coefficients from our model were close to .5, we found that that the inverse variance weighted approach with the standard correction produced effect size estimates similar to those from our meta-analytic model. In contrast, the uncorrected approaches produced substantially deflated effect size estimates. The Z Statistic approach, even with the standard correction, still tended to produce somewhat deflated effect size estimates. This can be attributed to the fact that the correction employed corrected for attenuation due to the indirect nature for the GWAX, but did not correct for variability in prevalence rates stemming from the ascertained nature of the samples.

## Discussion

Recent reports of very low SNP heritability of AD have called into question the prospects of continued progress in genetic discovery for AD within the spectrum of common variants. We have demonstrated that common methods used to produce these estimates are dramatically downwardly biased. We have introduced and validated a novel multivariate method for the joint analysis of direct GWAS and proxy GWAX summary data that relaxes standard assumptions and recovers unbiased estimates of common variant SNP heritability and of individual SNP effects under a variety of conditions. Compared to other naïve methods that boost power for estimating individual SNP effects on one phenotype by incorporating GWAS data from correlated phenotypes, our multivariate model specified within Genomic SEM is a formal model of multivariate genetic architecture from which both interpretable genome-wide parameters of interest, and individual SNP-specific effects can be estimated. Additionally, our model is unique in providing indices for violation of assumptions (e.g. Q_SNP_, see section 7 in S1 Supplementary Note), which may safeguard against cross-contamination of genome-wide signals across correlated traits.

Using recently released GWAS and GWAX summary data for AD, we find some modest deviations of the patterning of genetic sharing from that expected under the standard GWAX model. In particular, whereas the standard GWAX model assumes that genetic effects are attenuated by 50% (in regression units; i.e. by 75% in R^2^ units) relative to direct GWAS, we estimate somewhat greater attenuation: 54% (i.e. 1-.463) for maternal GWAX and 63% (i.e. 1-.366) for paternal GWAX. Interestingly, residual genetic variance specific to the individual GWAX was trivial, and there was no evidence of heterogeneity (Q_SNP_) of individual SNP effects beyond that expected based on our multivariate model. Taken together, these results suggest that GWAX for Clinical AD in UK Biobank may result in greater attenuation of genetic effects beyond that conventionally assumed, but does not introduce appreciable contamination of signal by genetically distinct traits relative to direct GWAS in IGAP.

Our multivariate method estimates the common variant liability-scale SNP heritability of AD excluding the *APOE* region to be more than double the recent estimate of 2.5% by Wightman et al. [4], when using same assumptions regarding population prevalence. This estimate rises to as high as 11% for Clinical AD and 23% for biological AD when considering prevalence rates indicated by collateral epidemiological and neuroimaging data. Of course, the estimated range of plausible values for the heritability of biological AD are based on assumptions regarding the extent to which the genetic signal represented in individuals diagnosed with clinical AD can be extrapolated to individuals with latent biological AD. Future research into the genetic architecture of biological AD will benefit from the large scale genomic analysis of direct measures of AD pathophysiology using, for example, positron emission tomography [10].

Analysis of local SNP heritability indicates that almost 90% of common variant SNP heritability of AD exists outside of the *APOE* region. We find that this remaining heritability accumulates relatively continuously across the remainder of the autosome, indicating that a portion of genetic risk for AD is characterized by a relatively diffuse polygenic architecture. This pattern resembles that recently observed for three molecular traits whose biological pathways are well-understood [24]. These authors inferred that core gene sets representing proximal biological mechanisms play a sizable role in each trait, but that “most of the SNP-based heritability is driven by a massively polygenic background.” Our results suggest that the same may pertain to the genetic architecture of AD, as has been generally postulated for complex traits by the omnigenic model [26].

Background polygenic architecture may reflect biological mechanisms underlying the pathophysiology of AD or ancillary factors that affect the likelihood of diagnosis of Clinical AD without contributing to disease onset or progression. The strongest candidates for such ancillary factors may be educational attainment and premorbid cognitive functioning, both of which longitudinal research indicates are not related to the onset or rate of cognitive decline but are related to the likelihood that declines will be detected by existing diagnostic protocols [7,27]. Genetic correlation analyses indicated that while these factors are indeed genetically correlated with AD, less than approximately 4% of SNP heritability of AD is explained by either factor, indicating that they are not by themselves sufficient to account for the polygenic component of AD genetic architecture. Longitudinal genomic research of neurocognitive change preceding and predicting eventual clinical AD will be of particular value for identifying pathways that may contribute to the polygenic risk for AD pathophysiology.

It is important to consider that the estimates of common variant SNP heritability from our multivariate modeling are based on the integration of summary data across many different cohorts. Between-cohort variability in the genetic architecture of AD across cohorts, and in methods for ascertainment and diagnosis can lead to attenuation of heritability during the aggregation process. Indeed, raw data based estimates of heritability using more homogeneous, yet smaller, cohorts [28] have often produced even larger heritability estimates than those reported here. This discrepancy could also be explained by the distribution of SNP effects of AD. If the core pathways for AD consist of a small number of causal variants with relatively large effects, then approaches that rely on assumptions of high polygenicity and normally distributed SNP effects may be yield underestimates of SNP heritability.

Our primary models relied on genetic covariance estimates derived using the standard LDSC model, which assumes homogeneous contributions to trait heritability across SNPs. Importantly, liability scale SNP heritability estimates continued to be sizable when alternative estimation methods were used. For instance, HESS allows for heritability to vary across different regions of the genome. The liability scale SNP heritability estimate obtained by applying HESS to the summary statistics produced by our multivariate model was 11.09% (9.92% excluding the *APOE* locus), assuming a population prevalence of 5%. Moreover, stratified LDSC allows for potentially uneven contributions of SNPs across biologically, evolutionarily, and MAF defined categories. When we fit our multivariate model to a genetic covariance matrix derived using stratified LDSC using 97 annotations, we obtained an estimate of liability scale SNP heritability of 8.94%, assuming a population prevalence of 5%. Importantly, we followed the standard practice of estimating genetic covariances using common variants. Rare variants not tagged in standard GWAS are likely to account for additional variance beyond the heritability estimates reported here. Whether the genetic covariance structure across GWAS and GWAX phenotypes differs for rare variants compared to common variants is an open question.

## Methods

### Bias in conventional meta analyses combining GWAS and GWAX

Recent large scale meta-analyses combining direct-GWAS and GWAX data have used either inverse variance weighted meta-analysis of regression coefficients [3,29] or sample size weighted meta-analysis of Z statistics [4,16,30]. For the inverse variance weighted approach, it is well-known that because of the indirect nature of the GWAX, the regression coefficients and associated standard errors (the squares of which represent the sampling variances used for inverse variance weighting) must be multiplied by a correction factor prior to meta-analysis [1]. The standard correction factor for GWAX of the phenotype of a single first degree relative using the offspring genotype is 2 to correct attenuation due to 50% genetically relatedness. Although not commonly implemented in the literature, we show in section 3 in S1 Supplementary Note that a correction must also be made when implementing the Z statistic approach. We show that for a continuous trait, the sample size must be divided by the square of the correction factor (e.g. by 4 for the standard correction) when implementing the Z statistic approach, with further corrections to sample size needed for case-control traits in ascertained samples. Naïve analysis of GWAX summary statistics produces SNP heritability estimates that are downwardly biased by 75% or more. This bias will carry forward in meta-analysis combining direct GWAS with GWAX statistics (see section 4 in S1 Supplementary Note and simulation below). This will occur when estimating SNP heritability from summary statistics even when regression coefficients and SEs have been corrected, because methods for estimating SNP heritability typically rely on the ratio of the regression coefficients to their standard errors (i.e. the Z statistics), which is preserved under the correction. We show that entering the effective sample size (obtained by dividing the observed N by the square of the correction factor) will produce the unbiased SNP heritability estimate when the appropriate correction factor is known (if the phenotype is a case-control trait, a conversion of the heritability estimate from the observed scale to the liability scale is still called for).

Whether the standard correction factor of 2.0 for GWAX regression coefficients is appropriate, and if not, how the appropriate correction factor can be identified is an unaddressed topic. The standard correction factor is derived simply on the basis of the 50% attenuation to regression estimates expected when proxy cases are first degree relatives of the genotyped individuals. However, attenuation will be more severe, such that the standard correction is insufficient, under a wide range of circumstances, such as those in which: a proportion of genotyped individuals report on the phenotypes of their step or adoptive parents (such that average genetic relatedness of phenotyped and genotyped individuals falls below 50%), when individuals are not well-informed about, misremember or confuse their parents’ phenotype or disease status (such that heritability of the GWAX phenotype is attenuated, or contaminated by other heritable phenotypes), or when the average quality of the diagnostics is of lower quality for parental history reports than for direct GWAS of carefully screened case-control sample (such that heritability of the GWAX phenotype is attenuated, or contaminated by other heritable phenotypes). We derive the expected attenuation bias to both regression coefficients and heritability and genetic covariance estimates analytically in section 3 and 4 in S1 Supplementary Note. Below, we introduce a data-driven multivariate approach for meta-analyzing GWAX and direct GWAS summary data that estimates the amount of attenuation in the GWAX (and thus the appropriate correction) directly from the data. Our approach formally models the genetic covariance structure of the GWAX and direct GWAS summary data in order to produce unbiased estimates of SNP heritability and individual SNP effects without manual correction of effect size estimates, standard errors, or sample sizes.

### A relaxed multivariate model for combining GWAS with GWAX

We use Genomic SEM to estimate a multivariate model of genetic risk for AD using summary data from three sources: direct GWAS, maternal GWAX, and paternal GWAX. In our multivariate model, the total genetic propensity toward AD risk is represented as a latent factor, *F*, that is specified to affect the direct GWAS phenotype and two GWAX phenotypes according to the following system of regression equations

$$\left[\begin{array}{c} Y_{direct}\\ Y_{mat}\\ Y_{pat} \end{array}\right]=\left[\begin{array}{c} \lambda_{direct}\\ \lambda_{mat}\\ \lambda_{pat} \end{array}\right]F+\left[\begin{array}{c} u_{direct}\\ u_{mat}\\ u_{pat} \end{array}\right],$$

or more compactly as

Y=ΛF+U,

where Λ is a vector of coefficients relating *F* to measured phenotypes *Y*, and U constitutes residual genetic propensities toward each of the measured phenotypes that are independent of *F*, and uncorrelated with one another and with *F*. This model is represented as a path diagram in S1 Fig (excluding the dashed portion).

This model implies that the genetic covariance matrix for *Y*_*Direct*_, *Y*_*Mat*_, and *Y*_*Pat*_ is given as

Σ=ΛΨΛ′+Θ,

where Ψ represents the covariances among the factors (and in this case contains a single element, representing the variance of F, i.e. $\sigma_{F}^{2}$), and Θ represents the covariances among the residuals, U (in this case a 3×3 diagonal matrix with diagonal elements $\sigma_{u_{Direct}}^{2},\;\sigma_{u_{Mat}}^{2}$, and $\sigma_{u_{Pat}}^{2}$).

We specify the model with the minimal identification constraint that *λ*_*direct*_ = 1 such that *F* takes on the scale of the direct GWAS phenotype, and $\sigma_{F}^{2}$ can be interpreted as an unbiased estimate of $h_{SNP}^{2}$ of the meta-analyzed phenotype in the direct GWAS metric. Under this parameterization the departure of *λ*_*mat*_ and *λ*_*pat*_ from .5 indicates departure of the empirical data from the standard GWAX model. We also consider an alternative parameterization in which we specify the model with the minimal identification constraint that $\sigma_{F}^{2}=1$, such that the variance of the latent factor F is standardized, and the freely estimated term *λ*_*Direct*_ can be interpreted as an unbiased estimate of $\sqrt{h_{SNP}^{2}}$ of the meta-analyzed phenotype in the direct GWAS metric. Under this parameterization, departure of *λ*_*mat*_/*λ*_*direct*_ and *λ*_*pat*_/*λ*_*direct*_ from .5 indicates departure of the empirical data from the standard GWAX model. Both parameterizations produce equivalent, just identified models, with 0 degrees of freedom, but differ in how parameters must be interpreted. These models can straightforwardly be extended to estimate genetic correlations between *F* and external GWAS phenotypes, such as educational attainment.

### Estimation of SNP effects for multivariate GWAS

The multivariate model can be expanded to include the effect of an individual genetic variant, *x*, on the latent factor, *F* (S1 Fig, including the dashed portion). Such a model comprises the following two sets of equations:

Y=ΛF+U,

F=γx+e,

where *γ* is an unstandardized regression coefficient and *e* is a residual. Such a model can be run for all available SNPs, one at a time, such that a complete set of meta-analytic summary statistics for *F* (in this case, AD), can be produced from the relaxed multivariate model. We use the minimal identification constraint *λ*_*direct*_ = 1, such that *γ* takes on the scale of the direct GWAS. To avoid the potential for variability in the optimization of the Λ and Θ parameters that may obscure interpretation of the *γ* coefficients across SNPs, we fixed these parameters to their values from the model without SNPs when estimating the model for each SNP in our empirical analysis. The resulting summary statistics from this multivariate GWAS serve as an alternative to those produced by more constrained Z statistic-based and (both corrected and uncorrected) inverse variance- based approaches. When contributing GWAS and GWAX are independent, as is the case for the empirical application presented here, the Effective N associated with the summary statistics from our multivariate model is calculated as Neff_Direct_ *+* Neff_Paternal_*λ_Paternal_^*2*^ *+* Neff_Maternal_*λ_Maternal_^*2*^, where the *λ*s are unstandardized factor loadings from the model. For summary statistics derived from a GWAS or GWAX meta-analysis, Neff = ∑4*v*_*k*_(1−*v*_*k*_)*n*_*k*_, and *v*_*k*_ is the sample prevalence for contributing study *k*. See Grotzinger et al. [31] for further detail.

### Model estimation

Models are estimated in Genomic SEM [18] using a two-stage approach. In the first stage, the empirical liability-scale genetic covariance matrix S and its sampling covariance matrix V are estimated using a multivariate version of LDSC. When the model to be fit includes individual SNP effects, the S matrix is expanded to include a vector of genetic covariances between the SNP and each of the GWAS and GWAX phenotypes that is derived directly from the univariate GWAS and GWAX estimates. The associated V matrix is also expanded using cross-trait intercepts from LDSC in order to take any potential sample overlap (known or unknown) and/or shared stratification implied by the LDSC model into account. In the second stage, the model is fit to the S matrix, and free parameters are estimated such that they minimize the discrepancy between Σ and S using the diagonally weighted least squares (WLS) fit function with sandwich correction, with weights derived from V, as described in Grotzinger et al. [18].

In a sensitivity analysis of the empirical data, we estimated the S matrix and its associated V matrix using stratified LDSC instead of LDSC, so as to allow for potentially uneven contributions of variants to heritability across biologically, evolutionarily, and MAF defined categories. Stratified LDSC was estimated with stratified Genomic SEM using 97 baseline annotations [21,32].

Note that the liability scale heritability is sensitive to assumptions about the population prevalence of AD. In our analyses of empirical data, our primary models set the population prevalence rate of AD at 5% used by Wightman et al. [4] to produce their estimate of liability-scale h^2^ of 2.5%., but we consider sensitivity of liability scale heritability estimates to other values, and to the possibility of undiagnosed AD within control participants (see Prevalence Rates in Relation to the Heritability of Clinical AD and AD Pathology, below). We also specifically compare estimates to those obtained with a population prevalence of 5% used by Wightman et al. [4] to produce their estimate of liability-scale h^2^ of 2.5%.

### Simulation study of SNP heritability

#### Simulation of summary statistics

We simulated genome-wide summary statistics for direct GWAS and maternal and paternal GWAX manipulating the attenuation coefficients (λ) for maternal and paternal GWAX assuming both continuous and binary traits. For binary traits we matched the sample prevalence for direct GWAS and maternal and paternal GWAX with those from IGAP (*v* = 0.344), UKB maternal (*v* = 0.096) and UKB paternal (*v* = 0.055), and a population prevalence of .05. S1 Table provides the details of the experimental design of our simulation study. For continuous traits, we provide six supplemental experimental conditions in which we additionally manipulated the sample size for the GWAX phenotypes compared with the direct GWAS (S10 Table). We simulated data for three phenotypes (direct GWAS, maternal GWAX, and paternal GWAX) per replication, and 100 replications per each of the conditions, for a total of 3,600 simulated sets of summary statistics. (As described below, each set of three summary statistics is then analyzed according to three meta-analytic methods, for a total of 3,600 meta-analyses). We used the parameter specifications provided in Table 1 to produce implied population-level genetic covariance matrices, Σ, calculated as

$$\left[\begin{array}{ccc} h_{1}^{2} & & \\ \sigma_{g1,2} & h_{2}^{2} & \\ \sigma_{g1,3} & \sigma_{g2,3} & h_{3}^{2} \end{array}\right]=\Lambda \Psi \Lambda^{\prime }=\left[\begin{array}{ccc} \lambda_{1}^{2}h_{F}^{2} & & \\ \lambda_{1}\lambda_{2}h_{F}^{2} & {\lambda_{2}^{2}h}_{F}^{2} & \\ \lambda_{1}\lambda_{3}h_{F}^{2} & \lambda_{2}\lambda_{3}h_{F}^{2} & {\lambda_{3}^{2}h}_{F}^{2} \end{array}\right],$$

Where $\Psi {=h}_{F}^{2}$ and Λ is the vector of attenuation coefficients (i.e. factor loadings, λ). We then used the LD scores for European population for M = 1,184,461 common Hapmap3 SNPs (MAF>.01 excluding the MHC region) provided by Bulik-Sullivan et al. [33], to simulate summary data for three phenotypes according to the multivariate LDSC equation, i.e.

$$[Z_{1j},Z_{2j},Z_{3j}]∼N({[0,0,0],cov(Z}_{1j},Z_{2j},Z_{3j})),$$

where

$${cov(Z}_{1j},Z_{2j},Z_{3j})=\left[\begin{array}{ccc} N_{1}\frac{h_{1}^{2}}{M}l(j)+1+a_{1} & & \\ \sqrt{N_{1}N_{2}}\frac{\sigma_{g1,2}}{M}l(j)+\frac{\rho_{12}N_{s12}}{\sqrt{N_{1}N_{2}}} & N_{2}\frac{h_{2}^{2}}{M}l(j)+1+a_{2} & \\ \sqrt{N_{1}N_{3}}\frac{\sigma_{g1,3}}{M}l(j)+\frac{\rho_{13}N_{s13}}{\sqrt{N_{1}N_{3}}} & \sqrt{N_{2}N_{3}}\frac{\sigma_{g23}}{M}l(j)+\frac{\rho_{23}N_{s23}}{\sqrt{N_{2}N_{3}}} & N_{3}\frac{h_{3}^{2}}{M}l(j)+1+a_{3} \end{array}\right]$$

and [*Z*_1*j*_, *Z*_2*j*_, *Z*_3*j*_] represents the *Z* statistics for the three GWAS/GWAX phenotypes, *N* is the sample size of the corresponding GWAS/GWAX, *M* is the number of SNPs including in the LD file, ℓ(j) is the LD score of SNP j (that is, the sum of squared correlations between the SNP and all other SNPs), N_s_ is the number of overlapping individuals in the corresponding GWAS/GWAX, *ρ* is the phenotypic correlation within the overlapping individuals, and α represents unmeasured sources of confounding such as population stratification.

We additionally simulated GWAS and GWAX summary data from a null scenario in which the model-implied genetic covariance matrix consisted of a diagonal matrix with heritabilities for direct, maternal, and paternal traits equal to .10, .025, and .025, respectively. In this scenario, genetic architecture is not shared across the GWAS and GWAX, and the multivariate heritability estimate is therefore expected to be 0. We fitted our multivariate Genomic SEM relaxed model on the simulated summary statistics to quantify Type I error rates for the model estimate of h^2^ across 100 replications.

Our simulation approach, in which summary statistics are directly generated from the LDSC equation is particularly well-suited to our purposes. Direct generation of summary statistics allows us to consider an expansive set of replications and conditions (i.e. 3,600 sets of simulated summary statistics total) that would be computationally prohibitive to simulate using a framework in which raw phenotype data were first generated for individual genomes and then submitted to GWAS. Summary data simulated under the LDSC model has the properties needed for analysis of genetic architecture by LDSC and for meta-analysis of effect sizes across phenotypes on a per-variant basis. Importantly, like any simulation approach, our approach also lacks some nuances of real data. For example, while we simulate summary data as a function of LD scores, we do not simulate summary data directly according to linkage disequilibrium (LD) structure (this would require the expansion of the covariance matrix of Z statistics across phenotypes to include cross-SNP covariances, which would dramatically increase computational burden). Thus, the simulated summary data are not appropriate for applications that are directly based on LD structure such as clumping and pruning, identification of lead SNPs within loci, or estimation of heritability using methods such as HDL [34] and HESS [23], which directly rely on LD structure (rather than simply on LD scores).

We present results of simulations in which we generate summary statistics under conditions of no sample overlap (*N*_*s*_ = 0) and no population stratification (*a* = 0). However, within this analytic framework, and in previous work using raw data simulation [18] we have confirmed that Genomic SEM produces unbiased estimates and standard errors when summary statistics are generated with sample overlap, phenotypic correlation within overlapping individuals, and population stratification.

### Analysis of simulated summary statistics

We compared the performance of three approaches to recover common variant observed SNP heritability for continuous traits and liability-scale heritability for binary traits: 1) LDSC of summary statistics from a conventional meta-analysis of Z statistics using the observed sample sizes (“Conventional Approach [Uncorrected]”), 2) LDSC of summary statistics from a conventional inverse-variance weighted meta-analysis of corrected betas and SEs assuming a simple GWAX model (“Conventional Approach [Standard Correction]”), and 3) our multivariate Genomic SEM-based approach, using the minimal identification constraint *λ*_*direct*_ = 1 such that $\sigma_{F}^{2}$ corresponds to the estimate of $h_{F}^{2}$). For binary traits we additionally introduced a correction for the conversion from observed to liability scale heritability (“Conventional Approach [Standard + Liability Correction]”). The liability correction consisted of using the sum of effective sample sizes (Neff, defined as 4*v*_*k*_(1−*v*_*k*_)*n*_*k*_, where *v*_*k*_ is the sample prevalence for study *k*) multiplied by the expected heritability attenuation based on a simple GWAX model (i.e., Neff_direct_+.25Neff_mat_+.25Neff_pat_). Because Neff represents the sample size for an equally balanced GWAS in terms of % of cases and controls (i.e., 50% cases, 50% controls), the sum of effective sample sizes is then entered in the LDSC stage, along with a .5 as sample prevalence. See Grotzinger et al. [31] for a more detailed explanation on the liability-scale correction based on the sum of effective sample sizes. The conventional uncorrected approach is reflective of the approaches taken by Jansen et al. [16] and Wightman et al. [4]. The conventional corrected approach is reflective of the approaches taken by Marioni et al. [3] and Bellenguez et al. [5]. The conventional approach with standard and liability correction combines the approaches taken by Marioni et al. [3] and Bellenguez et al. [5] with the liability-scale correction introduced by Grotzinger et al. [31], thus maintaining the standard GWAX assumptions while eliminating bias arising from differences in ascertainment across the GWAS and GWAXs.

For each condition, we calculated: 1) mean parameter estimate, 2) standard deviation (SD) of the parameter estimate, 3) mean standard error (SE) of the parameter estimate, and 4) Percent Bias Error (%BE) of the parameter estimate, calculated as:

$$\%BE=\frac{1}{100}\sum_{r=1}^{100}\left(\frac{\hat{h_{l,r}^{2}}}{h_{l,True}^{2}}-1\right)$$

Where $\hat{{h_{l}^{2}}_{r}}$ is the parameter estimate in replication *r*, and $h_{l,True}^{2}$ is its true value for that particular condition.

### Simulation study of individual SNP effects

We extended our simulations for recovery of heritability to allow us to test for the recovery of individual SNP effects for both continuous and binary traits. Although the simulation for recovery of heritability generated individual level SNP effects, those analyses simulated based on a population model with a known genetic covariance structure, but not known effects for individual SNPs. However, in order to benchmark performance of each method for estimating individual SNP effects we must know their true effect size in the population from which we draw our simulation. We therefore extended the simulation analyses to include a single individual SNP per replication with a known true effect according to the conditions in S1 Table. We set the true effect (*γ*) of this SNP on *F* to be equal to the partially standardized (i.e. standard deviations in liability for AD per effect allele) beta coefficient of genome-wide-significant SNP rs60738304 from the IGAP summary statistics (*b* = .068, MAF = 0.305), such that

$$[b_{1j},b_{2j},b_{3j}]=\gamma \Lambda =[.068\lambda_{1},.068\lambda_{2},.068\lambda_{3}].$$

For continuous traits, we sampled observed regression coefficients from their sample-size dependent sampling distribution as follows

$$[\hat{b_{1}},\hat{b_{2}},\hat{b_{3}}]∼N\left([b_{1},b_{2},b_{3}],[\begin{array}{ccc} \frac{1-{{2MAF(1-MAF)b}_{1}}^{2}}{{2MAF(1-MAF]n}_{1}} & & \\ 0 & \frac{1-{{2MAF(1-MAF)b}_{2}}^{2}}{{2MAF(1-MAF)n}_{2}} & \\ 0 & 0 & \frac{1-{2MAF(1-MAF)b_{3}}^{2}}{{2MAF(1-MAF)n}_{3}} \end{array}]\right),$$

For binary traits, the regression coefficients were sampled from their sampling distribution as:

$$[\hat{b_{1}},\hat{b_{2}},\hat{b_{3}}]∼N\left([b_{1},b_{2},b_{3}],[\begin{array}{ccc} \frac{1-{{2MAF(1-MAF)b}_{1}}^{2}}{{2MAF(1-MAF)v(1-v)n}_{1}} & & \\ 0 & \frac{1-{{2MAF(1-MAF)b}_{2}}^{2}}{{2MAF(1-MAF)v(1-v)n}_{2}} & \\ 0 & 0 & \frac{1-{2MAF(1-MAF)b_{3}}^{2}}{{2MAF(1-MAF)v(1-v)n}_{3}} \end{array}]\right)$$

Note that because we simulate under conditions of no sample overlap across any of the direct GWAS and two GWAX datasets, the sampling covariance matrix of the coefficients is diagonal in the population model (the corresponding cells in the V matrix are nevertheless freely estimated when the data are analyzed in Genomic SEM, such that any sample overlap that does exist is automatically detected and corrected for).

### Analysis of simulated data

We computed meta-analytic estimates of *γ* using the conventional corrected and uncorrected approaches, and the multivariate Genomic SEM approach (model with individual SNP effects using the minimal identification constraint *λ*_*direct*_ = 1, such that *γ* takes on the scale of the direct GWAS). Because the Genomic SEM approach requires a genetic covariance matrix that includes terms for individual SNP effects and terms for genome-wide genetic covariances (the genome-wide covariance structure informs the estimation of individual SNP effects), we converted the simulated regression coefficients to SNP-level genetic covariances and appended them to genome-wide genetic covariance matrices estimated from LDSC using the genome-wide summary statistics produced under the simulation of SNP heritability for the corresponding condition.

For each simulation condition, we calculated: 1) mean parameter estimate, 2) standard deviation (SD) of the parameter estimate, 3) mean standard error (SE) of the parameter estimate, 4) mean Z statistic of the parameter estimate, and 5) Percent Bias Error (%BE) of the parameter estimate.

### Selection of GWAS and GWAX summary data

We compiled summary data from three published European ancestry direct case-control GWAS of AD and GWAX of parental history of AD.

Direct case-control GWAS summary statistics encompassed the discovery sample from the IGAP consortium [12] comprising 21,982 Clinical AD cases (mean age of onset = 72.93 years) and 41,944 controls (mean age of evaluation = 72.415 years). Cohorts contributing to IGAP varied considerably in the extent to which clinical determination of case and control status was confirmed by autopsy. To avoid bias otherwise arising from variation in case prevalence across cohorts contributing to IGAP, we followed the approach described by Grotzinger et al. [31], using the sum of effective sample sizes (4*v*_*k*_(1−*v*_*k*_)*n*_*k*_, where *v*_*k*_ is the sample prevalence for each GWAS contributing to the GWAS meta-analysis). Our GWAX summary data of proxy-phenotype AD included 27,696 cases and 260,980 controls of history of maternal AD, and 14,338 cases and 245,941 controls of history of paternal AD, both phenotypes from UK Biobank [3]. Case status for UK Biobank was determined by response to the following two questions “Has/did your father ever suffer from Alzheimer’s disease/dementia?” and “Has/did your mother ever suffer from Alzheimer’s disease/dementia?” at the initial assessment visit (2006–2010), the first repeat assessment visit (2012–2013) and the imaging visit (2014+). Participants whose parents were younger than 60 years or died prior to age 60 years, and without parental age information were excluded. Zhang et al. [28] report that the mean age of maternal cases was 83.7 years, the mean age of paternal cases was 81.8 years, the mean age of maternal controls was 78.1 years, and the mean age of paternal controls was 76.2 years. Further details on case ascertainment, genotyping, and quality control can be found in the original articles for the corresponding summary statistics.

We additionally curated the following GWAS summary statistics for the estimation of genetic correlations: two recent meta-analyses of direct GWAS and GWAX of AD [3,16], brain volume [35] in the general population, educational attainment [36] in the general population and a general genetic factor of cognitive function [37] in the general population.

### Quality control of GWAS and GWAX summary aata

#### LD-Score Regression (LDSC)

All summary statistics used for LDSC were cleaned and processed using the munge function of Genomic SEM, using the standard practice of retaining all HapMap3 [38] SNPs outside of the major histocompatibility complex (MHC) region with minor allele frequencies (MAFs) ≥ .01 and information scores (INFO) > .9. To avoid misfit in LD score regression due to extremely large effect sizes within the *APOE* region, we additionally remove this region (CHR19:45,116,911–46,318,605). This has a similar effect to the standard practice in LDSC of removing SNPs with extremely large test statistics. The LD scores used for the analyses presented were estimated from the European sample of 1000 Genomes, but restricted to HapMap3 SNPs as these tend to be well-imputed and produce accurate estimates of heritability. In the LDSC equation, we enter the total number of SNPs in the reference LD panel before excluding the MHC and *APOE* regions, such that the SNP heritability estimates reported here retain similar interpretation to those typically produced by LDSC in other contexts. Based on these procedures, the SNP heritability estimates produced by LDSC should be considered estimates of heritability explained by common SNPs, not including the extremely strong effect of *APOE*.

### Multivariate GWAS

For the SNP portion of the multivariate model that included individual SNP effects, we used the default QC procedures in Genomic SEM [18] of removing SNPs with an MAF < .005 in the 1000 Genomes Phase 3 reference panel and SNPs with an INFO score < 0.6 in the univariate GWAS summary statistics. Using these QC procedures left 7,192,577 SNPs across the three contributing summary statistics datasets. Prior to running any multivariate GWAS, all SNP effects were converted to logistic regression coefficients, standardized with respect to the total liability scale variance in the outcome using the sumstats function in GenomicSEM, and corrected for genomic inflation by multiplying the standard errors by square root of the univariate LDSC intercept when the intercept was above 1. These transformed estimates were then multiplied by the SNP variance (estimated as 2*MAF(1-MAF)) to produce genetic covariances. In the case of some variants within the *APOE* region with very large effects, we reduced the MAF relative to its reference panel value when calculating the SNP variance in order to prevent the full S matrix (containing both SNP effects and genetic covariances among the direct GWAS and two GWAX phenotypes) from being nonpositive definite. As we ultimately estimate SNP effects in per effect-allele units, rather than in per standard deviation units, this decision does not bias the estimates of interest.

### Post GWAS analysis of meta-analytic results in FUMA

We applied FUMA [39] to results of our multivariate GWAS meta-analysis to identify independent significant SNPs, lead SNPs, and risk loci, using the defaults. Independent significant SNPs were defined as genome wide significant SNPs that are independent from one another at *r*^2^<.60. Independent significant SNPs are clumped to identify lead SNPs, which are independent from each other at r^2^ < 0.1. In defining genomic risk loci, independent significant SNPs that are associated *r*^2^≥.10 are assigned to the same locus, and independent significant SNPs closer than 250kb are merged into a single locus. The most significant lead SNP in a locus is used to represent that locus. The EUR population from 1000 Genomes Phase 3 was used as the reference panel. As 16 SNPs in the *APOE* block (rs429358) produced very large Z statistics such that their p-value was treated by FUMA as 0 and excluded, we manually entered the most significant SNP in the *APOE* block into FUMA as the lead SNP.

### Prevalence rates in relation to the heritability of clinical AD and biological AD

#### Clinical AD

When methods for estimating SNP heritability, such as LDSC, are applied to GWAS of binary phenotypes, the estimate of heritability are on the *observed scale*. Observed scale heritability is difficult to interpret, because it takes on a metric that is idiosyncratic to the balance of cases and controls in the sample, and agnostic to the population prevalence rates of the categories. It is more interpretable to convert observed scale heritability to liability scale heritability based on a liability threshold model, which assumes that a normally distributed liability toward the binary phenotype exists in a population. Under this model, individuals with a liability above a certain threshold are affected whereas those below this threshold are unaffected. The liability-scale SNP heritability estimate is therefore an estimate of the proportion of variation in this continuous liability that is explained by all (directly and indirectly tagged) SNPs on which the GWAS is based. For ascertained samples, in which the proportion of cases does not represent their prevalence in the population, and assuming that the control participants are successfully screened, such that they are unaffected by the disorder (an issue that we return to next), the expected liability scale heritability (${\hat{h}}_{l}^{2}$) can be obtained from observed scale heritability ($h_{o}^{2}$) according to Peyrot et al. [40]

$${\hat{h}}_{l}^{2}=\frac{P_{population}^{2}{\left(1-P_{population}\right)}^{2}}{P_{sample}(1-P_{sample}){\varphi (t)}^{2}}h_{o}^{2},$$

where *P*_*sample*_ is the proportion of cases in the GWAS sample, *P*_*population*_ is the proportion of cases in the population (i.e. the population prevalence), *t* is the threshold of the cumulative normal distribution corresponding to the population prevalence, and *φ*(*t*) is the density of the normal distribution at *t*.

Whereas *P*_*sample*_ is known, *P*_*population*_ must be obtained from collateral epidemiological data. As Yang et al. [41] write, **“**Application of [the transformation from observed scale heritability to liability scale heritability] requires an estimate of [the population prevalence of the disorder], for which estimates can be surprisingly hard to find, and most applications include a sensitivity analysis to the choice of [this parameter].” Following this guidance, we provide estimates of liability scale heritability across a range of different prevalence rates, and provide rough age equivalents of these prevalence rates based on published epidemiological data from the Medical Research Council Cognitive Function and Ageing Study II [19]. We refer to this estimate as the estimate of heritability of Clinical AD, in that this is the estimate based on the prevalence rate of the clinical diagnosis, and does not account for undetected biological AD that may be more prevalent in the population.

### Biological AD

The past decade of research on AD has been increasingly cognizant of the fact that “the pathophysiological process of [AD] begins years, if not decades, before the diagnosis of dementia” [42]. Those presenting with Clinical AD may not constitute those individuals with the most severe forms of AD pathophysiology, but may be constitute a group whose cognitive impairments are more overt or detectable due to ancillary characteristics (such as those related to peak premorbid levels of cognitive functioning) that are unrelated to the genetics of biological AD [27]. Indeed, this common observation that differences in ancillary factors such as educational attainment are related to differences in Clinical AD rates among individuals with equivalent levels of brain pathology has led to the popular concept of *cognitive reserve* [7,20,43]. In order to estimate the heritability of biological AD, we capitalize on the Clinical AD GWAS and the age-specific rates of biological AD. We employ the formula for converting observed to liability scale heritability that corrects for case contamination in control participants provided by Peyrot et al. [40], further adapting to allow for imperfect screening of participants (i.e. screening for Clinical but not biological AD):

$${\hat{h}}_{l}^{2}=\frac{P_{population\;Biological\;AD}^{2}{\left(1-P_{population\;Biological\;AD}\right)}^{2}}{P_{sample\;Clinical\;AD}(1-P_{sample\;Clinical\;AD}){\left(1-FCR\right)}^{2}{\varphi (t)}^{2}}h_{o}^{2},$$

where *FCR* is the false classification rate: the proportion of incorrectly classified control participants in the GWAS. When control participants are a random sample of the population, we set *FCR* = *P*_*population*_. However, because controls have been screened for clinical AD but not biological AD, and we estimate the liability scale heritability for biological AD under the assumption that clinical AD represents a random subset of biological AD, and compute *FCR* as

$${FCR=(P}_{population\;Biological\;AD}-P_{population\;Clinical\;AD})$$

We provide estimates of liability scale heritability of biological AD according to this equation across a range of different population prevalence rates for biological AD, and provide rough age equivalents of these prevalence rates based on recently published data from Jack et al. [10]. To achieve this, we fix *P*_*population Clinical AD*_ at the approximate age-specific estimate of the clinical AD prevalence rate from epidemiological data for the mean age of the control sample in IGAP (72.42 years, prevalence rate = 0.0285) to reflect the expected proportion of participants that have been screened out of the control sample for clinical AD) and we vary *P*_*population Biological AD*_.

### Local SNP heritability in HESS

We used HESS to estimate local common variant SNP heritability in 1,703 approximately independent blocks across the genome, using common variants (MAF>.01) in the EUR population from 1000 Genomes as the reference panel [23]. Because LDSC relies on the variation in average LD across SNPs to estimate SNP heritability, it cannot be used to estimate local heritability within individual loci in which LD is relatively homogeneously high. In contrast, because HESS relies on the LD matrix itself to estimate SNP heritability, and does not specifically rely on variation in average LD, it is more appropriate for estimating local heritability.

## Supporting information

S1 Supplementary Note Integrated Analysis of Direct and Proxy Genome Wide Association Studies Highlights Polygenicity of Alzheimer’s Disease outside of the APOE Region. (DOCX)

S2 Supplementary Results Integrated Analysis of Direct and Proxy Genome Wide Association Studies Highlights Polygenicity of Alzheimer’s Disease outside of the APOE Region. (DOCX)

S1 Fig Path diagram for multivariate genetic analysis of Alzheimer’s disease. We present the minimal identification constraint *λ*_*direct*_ = 1 such that the variance of the factor corresponds to the meta-analytic heritability estimate on scale of the direct GWAS. The dashed portion of the diagram represents the portion of the model that is specified to produce meta-analytic estimates for effects of individual SNPs.
(TIF)

S2 FigSimulation Results.Distribution of observed SNP heritability estimates ($h_{SNP}^{2}$) across supplemental conditions S1-S6 for continuous traits.(TIFF)

S3 Fig Simulation Results. Distribution of individual SNP effects across supplemental conditions S1-S6 for continuous traits.
(TIFF)

S4 Fig Heatmaps of LDSC cross-trait intercepts (left) and genetic correlations (right) among direct GWAS, maternal and paternal GWAX, and meta-analytic summary statistics of Alzheimer’s disease from two previous studies, and from the Genomic SEM-based multivariate method introduced here.
(TIFF)

S5 Fig Manhattan plot for the common factor GWAS of Alzheimer’s disease. *Note*: the upper limit for the y axis was constrained to 50, the APOE region goes off-scale (lead SNP rs429358 -log_10_(p) = 308.653).
(TIFF)

S6 Fig QQ-plot for AD Genomic SEM relaxed model and heterogeneity Q. Mean χ^2^ for AD GSEM = 1.139. Mean χ^2^ for heterogeneity Q = 1.008.
(TIF)

S7 Fig Estimated common variant SNP heritability of AD (outside of the MHC and *APOE* regions) according to different assumptions regarding the population prevalence of AD and biological AD, based on LDSC of summary statistics from our multivariate meta-analysis. We provide rough approximations of the age equivalences of each prevalence rate on the top x axis. The purple diamond represents the estimate of 2.5% by Wightman et al.[4], which was based on an assumed population prevalence rate of 5%. The yellow diamond represents the estimate from the multivariate model introduced here, using the same assumed population prevalence rate of 5% (Clinical AD h^2^ = 0.069; Biological AD h^2^ = 0.072). The shading area around the line reflects +/- 1 SE of the h^2^ estimate. The steeper shift in the SNP heritability of AD for biological AD compared to clinical AD as a function of population prevalence stems from the correction for undetected biological AD within control participants who primarily only been screened for clinical AD. Thus, as the assumed prevalence rate of biological AD increases, the extent of case contamination in control participants increases, and the correction for undetected AD in control participants produces more dramatic increases in the projected heritability.
(TIFF)

S8 Fig Association between local SNP heritability and GWAS effect sizes for GWAS loci that were genome wide significant in the multivariate GWAS including the APOE locus containing lead SNP rs429358 (left) and excluding this locus (right).
(TIFF)

S9 Fig Local SNP heritability results for IGAP. (TIFF)

S10 Fig Heatmap of LDSC genetic correlations among meta-analyses of case-control and proxy-phenotype family history of Alzheimer’s disease and external correlates of Alzheimer’s disease. (TIFF)

S1 Table Experimental conditions for the simulation of summary statistics and SNP effect sizes for binary traits. (XLSX)

S2 Table Recovery of SNP-based heritability across experimental conditions and methods (100 runs per condition). (XLSX)

S3 Table Recovery of individual SNP effects across experimental conditions and methods (100 runs per condition). (XLSX)

S4 Table Genome-wide significant (P < 5 x 10–8) SNP association results for Genomic SEM AD. (XLSX)

S5 Table Genome-wide significant risk loci associated with Genomic SEM AD. (XLSX)

S6 Table Effect sizes associated with lead SNPs of genomic risk loci across contributing datasets and meta-analytic approaches. (XLSX)

S7 Table Lead SNPs and tagged SNPs for GSEM AD previously identified in GWAS of diseases and traits listed in the GWAS catalog. (XLSX)

S8 Table Independent lead SNPs and SNPs tagged within each significant genomic locus for GSEM AD. (XLSX)

S9 Table Type I error rates for heritability estimates simulated from a null model (100 iterations). (XLSX)

S10 Table Supplemental experimental conditions for the simulation of summary statistics and SNP effect sizes for continuous traits. (XLSX)

S11 Table Recovery of SNP-based heritability across supplemental experimental conditions and methods for continuous traits (100 runs per condition). (XLSX)

S12 Table Recovery of individual SNP effects across supplemental experimental conditions and methods for continuous traits (100 runs per condition). (XLSX)
