# Supplementary material for: Integrated analysis of direct and proxy genome wide association studies highlights polygenicity of Alzheimer’s disease outside of the APOE region
Source: PLoS Genet. 2022 Jun 3;18(6):e1010208. doi: 10.1371/journal.pgen.1010208 (PMC9200312; doi:10.1371/journal.pgen.1010208)
Supplement: S2 Supplementary Results — (DOCX) [file pgen.1010208.s002.docx]

**SUPPLEMENTARY RESULTS**

**Integrated Analysis of Direct and Proxy Genome Wide Association Studies Highlights**

**Polygenicity of Alzheimer’s Disease outside of the APOE Region**

Javier de la Fuente,

Andrew D. Grotzinger, Riccardo E. Marioni, Michel G. Nivard,

& Elliot M. Tucker-Drob

**SR1. Genetic Correlations with previous GWAS Meta-Analyses of Alzheimer’s Disease and other External Correlates**

Fig S4 provides LDSC intercepts and LDSC-estimated genetic correlations of our multivariate AD meta-analysis with those of Marioni et al. and Jansen et al. with one another and with the direct GWAS of AD in IGAP and the GWAXs of maternal and paternal AD in UKB. It can be seen that the genetic correlations of the three meta analyses all exceed 1.0, indicating that the same genetic signal is tapped by each of them. The cross-trait intercepts are also very high (.65-.87) for the pairwise combinations of the three meta-analyses, as expected from their reliance on largely the same data. Investigating the genetic correlations between each of the three meta-analyses and the direct GWAS and two contributing GWAX, it can be seen that the Marioni and Jansen summary statistics demonstrate some highly out-of-bound associations (i.e. r_g_ of 2.03 between Marioni meta analysis and IGAP; and r_g_ of 1.80 and 1.73 between the Jansen meta-analyses and UKB maternal and paternal GWAX, respectively). In contrast, the associations between those produced by our multivariate method within Genomic SEM and the direct GWAS and two contributing GWAX are less extreme (e.g. the only out-of-bond estimated is the r_g_ of 1.16 between the multivariate meta-analysis and IGAP). It is possible that these differences stem from the differences in whether or not the optimal weights were used in the respective meta-analyses.

Fig S10 provides LDSC-estimated genetic correlations of our multivariate AD meta-analysis, and those by Marioni et al. and Jansen et al. with brain volume, educational attainment, and general cognitive function in the general population. AD risk, as indexed by Jansen et al. meta-analysis was more strongly genetically correlated with educational attainment (r_g_=-.2) than was AD risk as indexed by the Marioni meta analysis (r_g_=-.07) and the multivariate meta analysis (r_g_=-.06). The three meta-analyses were consistently related to a general genetic factor of cognitive function (r_g_ = -.20 for our multivariate meta-analysis, and r_g_ = -0.19 for the meta-analyses by Marioni and Jansen). There were no meaningful genetic associations with brain volume. Note that all LDSC analyses were based on common variants (MAF ≥ .01) outside of the MHC and APOE regions. Because other work has indicated very little evidence for genetic correlations between AD and other GWAS traits, we did not examine a wider range of genetic correlates.
