# Supplementary figures and images for: Integrated analysis of direct and proxy genome wide association studies highlights polygenicity of Alzheimer’s disease outside of the APOE region
Source: PLoS Genet. 2022 Jun 3;18(6):e1010208. doi: 10.1371/journal.pgen.1010208 (PMC9200312; doi:10.1371/journal.pgen.1010208)

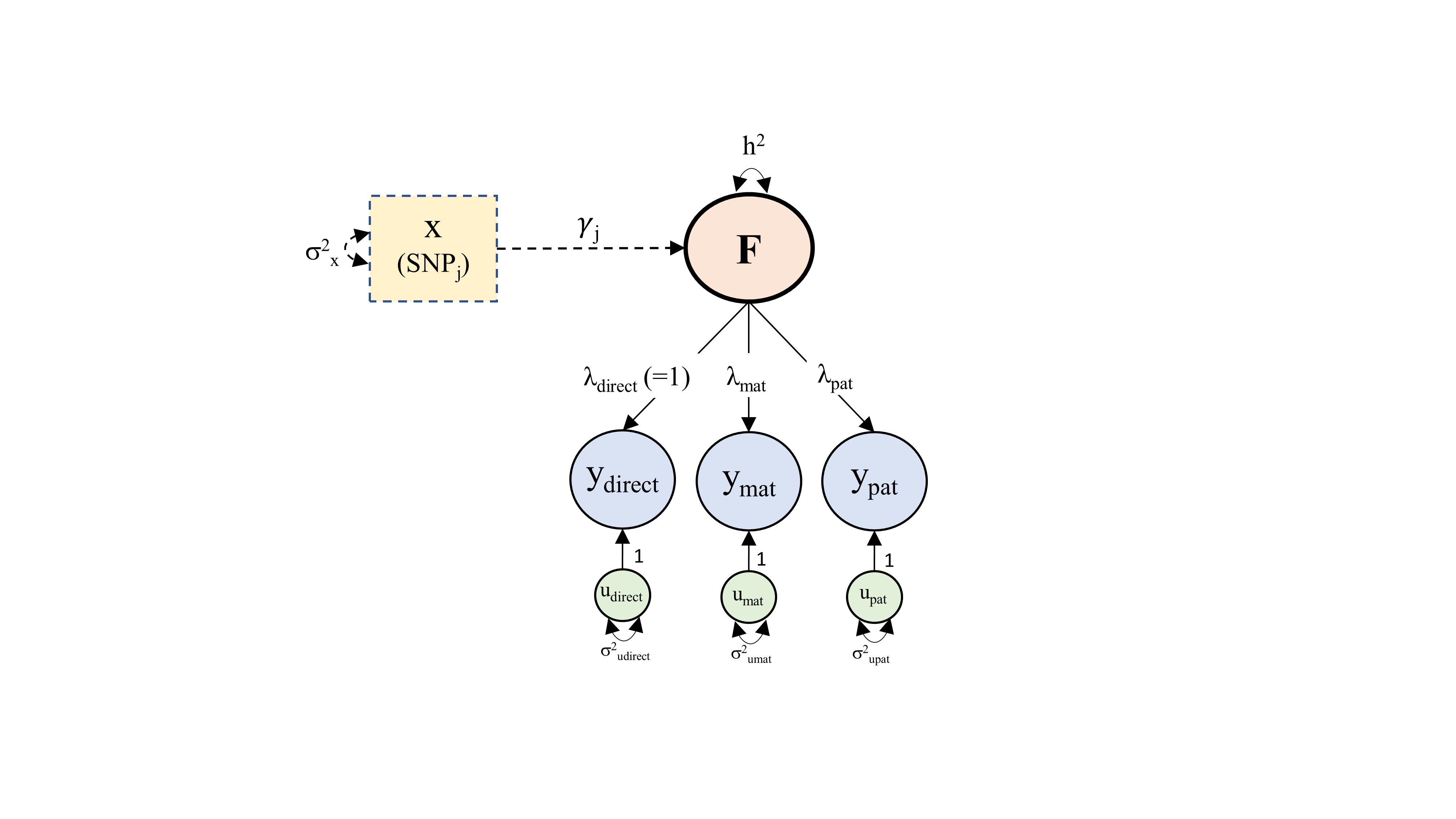

Supplement: S1 Fig — We present the minimal identification constraint λdirect = 1 such that the variance of the factor corresponds to the meta-analytic heritability estimate on scale of the direct GWAS. The dashed portion of the diagram represents the portion of the model that is specified to produce meta-analytic estimates for effects of individual SNPs. (TIF) [file pgen.1010208.s003.tif]

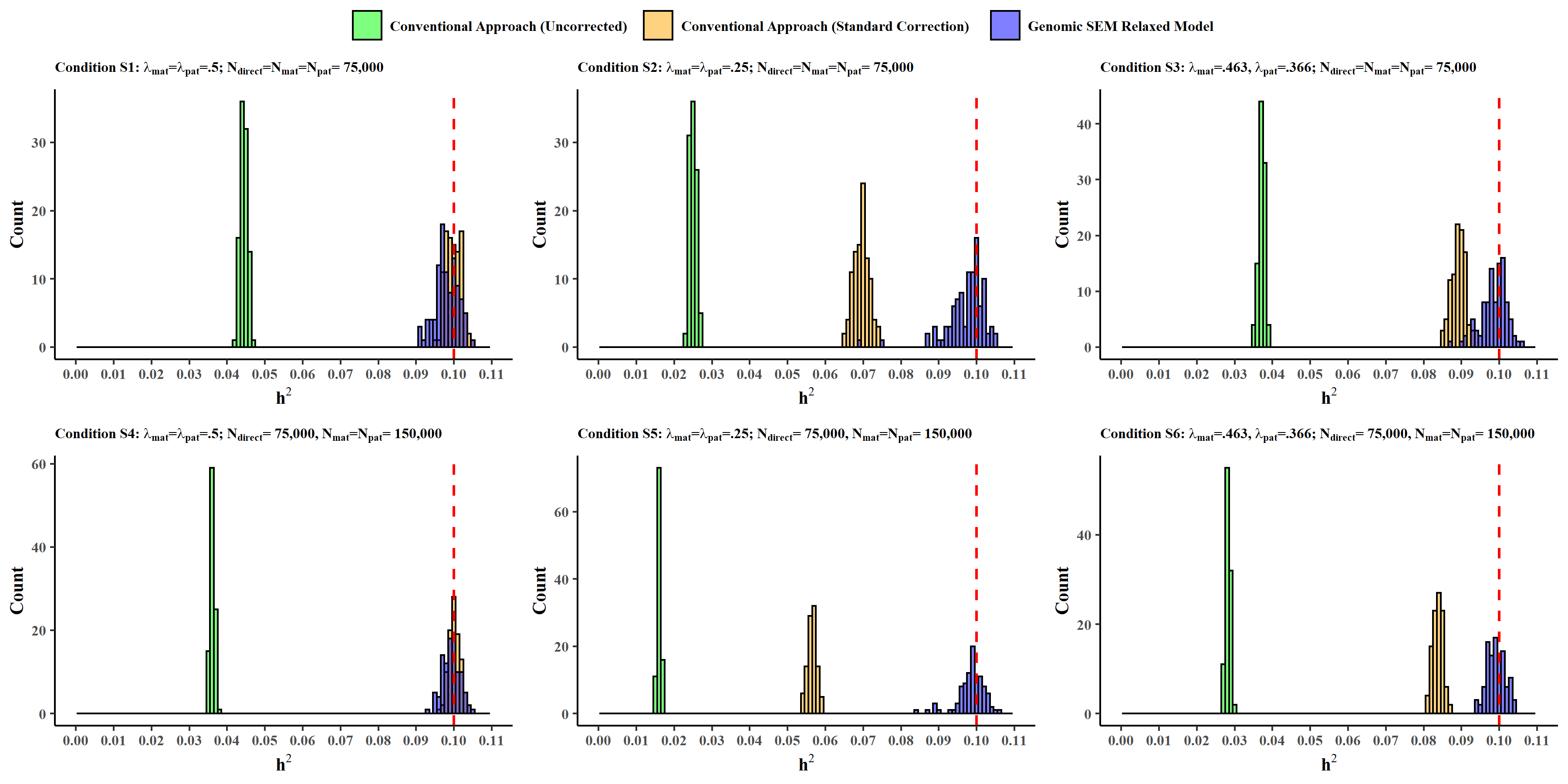

Supplement: S2 Fig — Distribution of observed SNP heritability estimates (hSNP2) across supplemental conditions S1-S6 for continuous traits. (TIFF) [file pgen.1010208.s004.tiff]

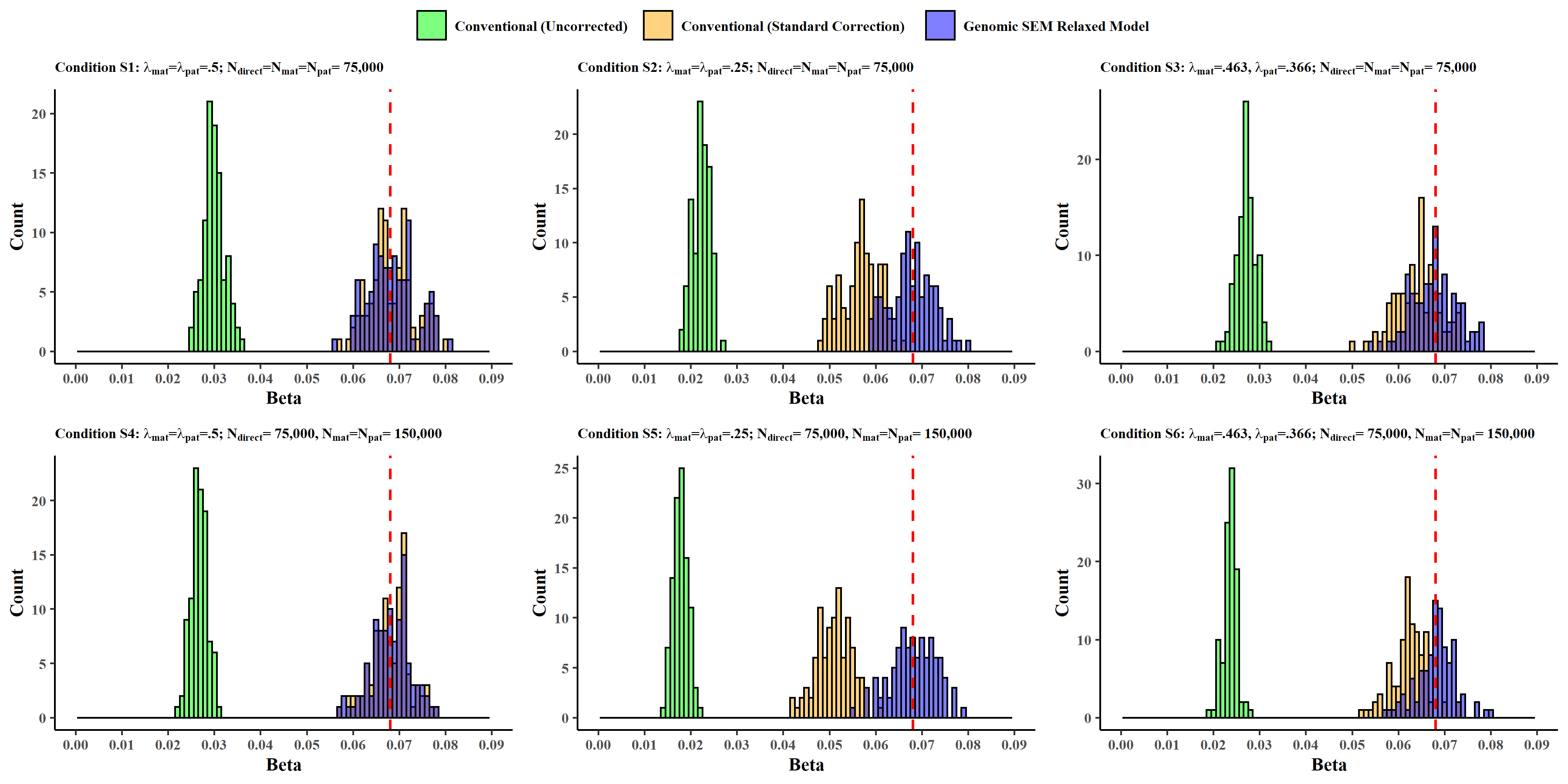

Supplement: S3 Fig — Distribution of individual SNP effects across supplemental conditions S1-S6 for continuous traits. (TIFF) [file pgen.1010208.s005.tiff]

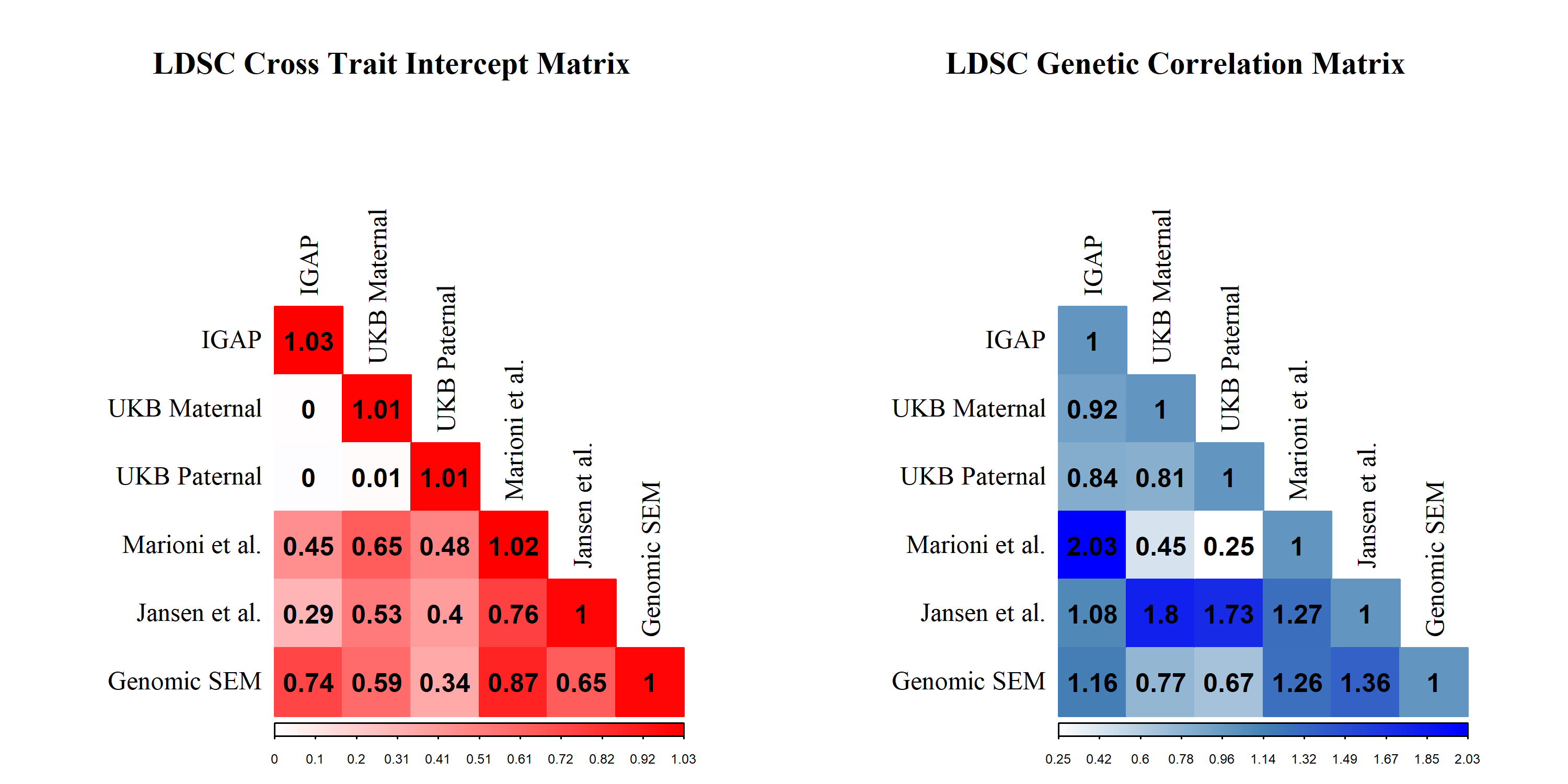

Supplement: S4 Fig — Heatmaps of LDSC cross-trait intercepts (left) and genetic correlations (right) among direct GWAS, maternal and paternal GWAX, and meta-analytic summary statistics of Alzheimer’s disease from two previous studies, and from the Genomic SEM-based multivariate method introduced here. (TIFF) [file pgen.1010208.s006.tiff]

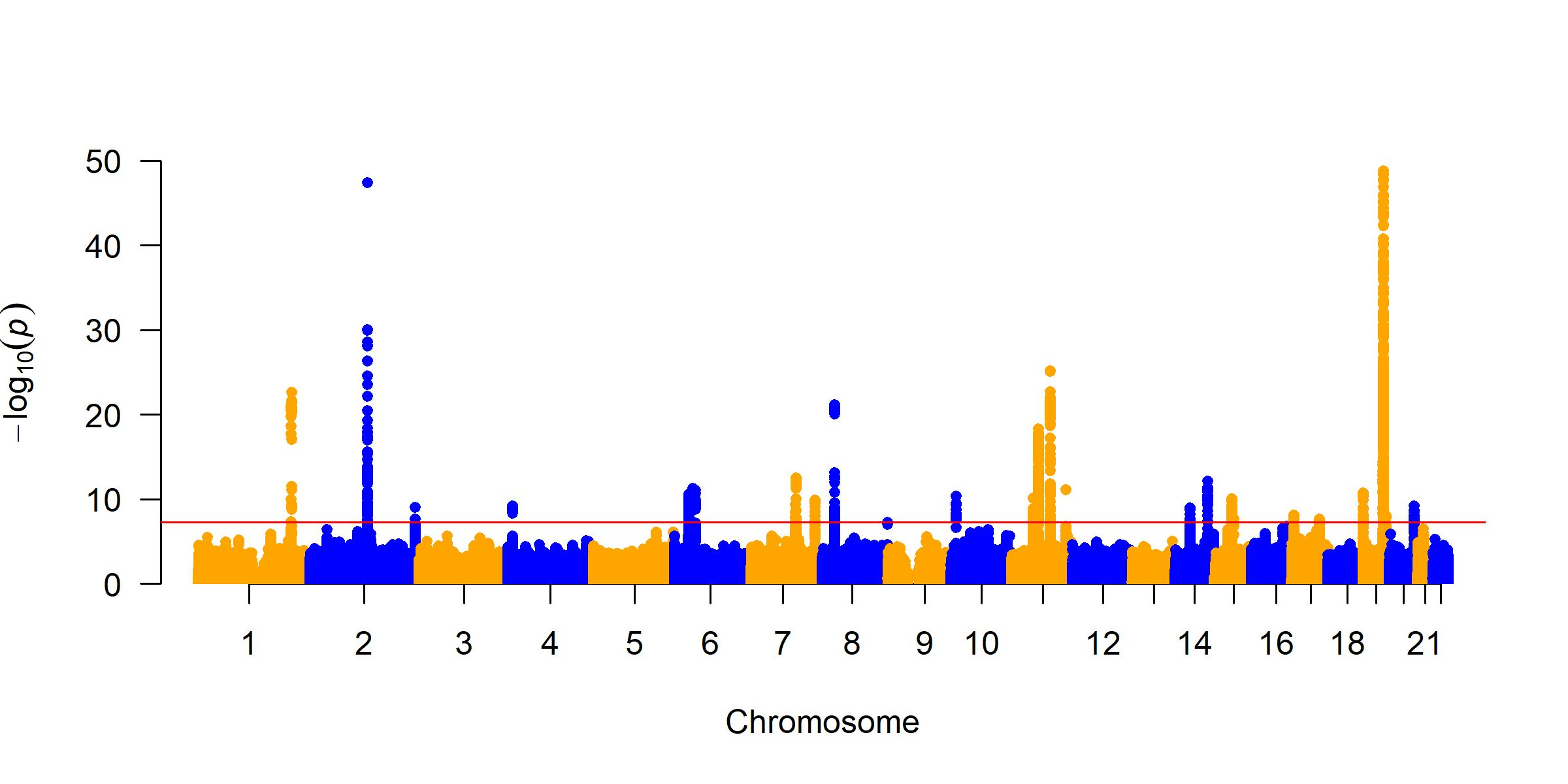

Supplement: S5 Fig — Note: the upper limit for the y axis was constrained to 50, the APOE region goes off-scale (lead SNP rs429358 -log10(p) = 308.653). (TIFF) [file pgen.1010208.s007.tiff]

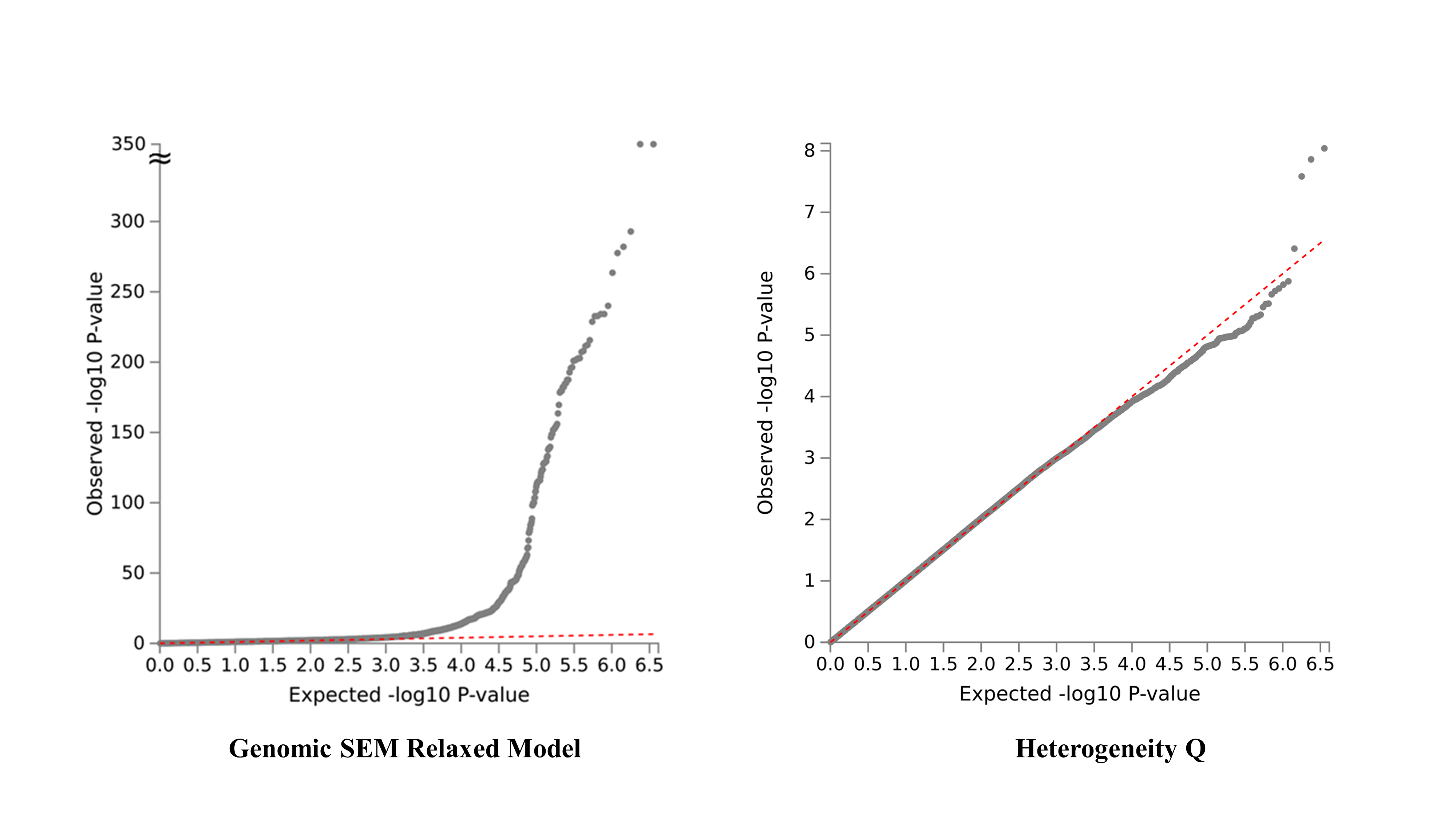

Supplement: S6 Fig — Mean χ2 for AD GSEM = 1.139. Mean χ2 for heterogeneity Q = 1.008. (TIF) [file pgen.1010208.s008.tif]

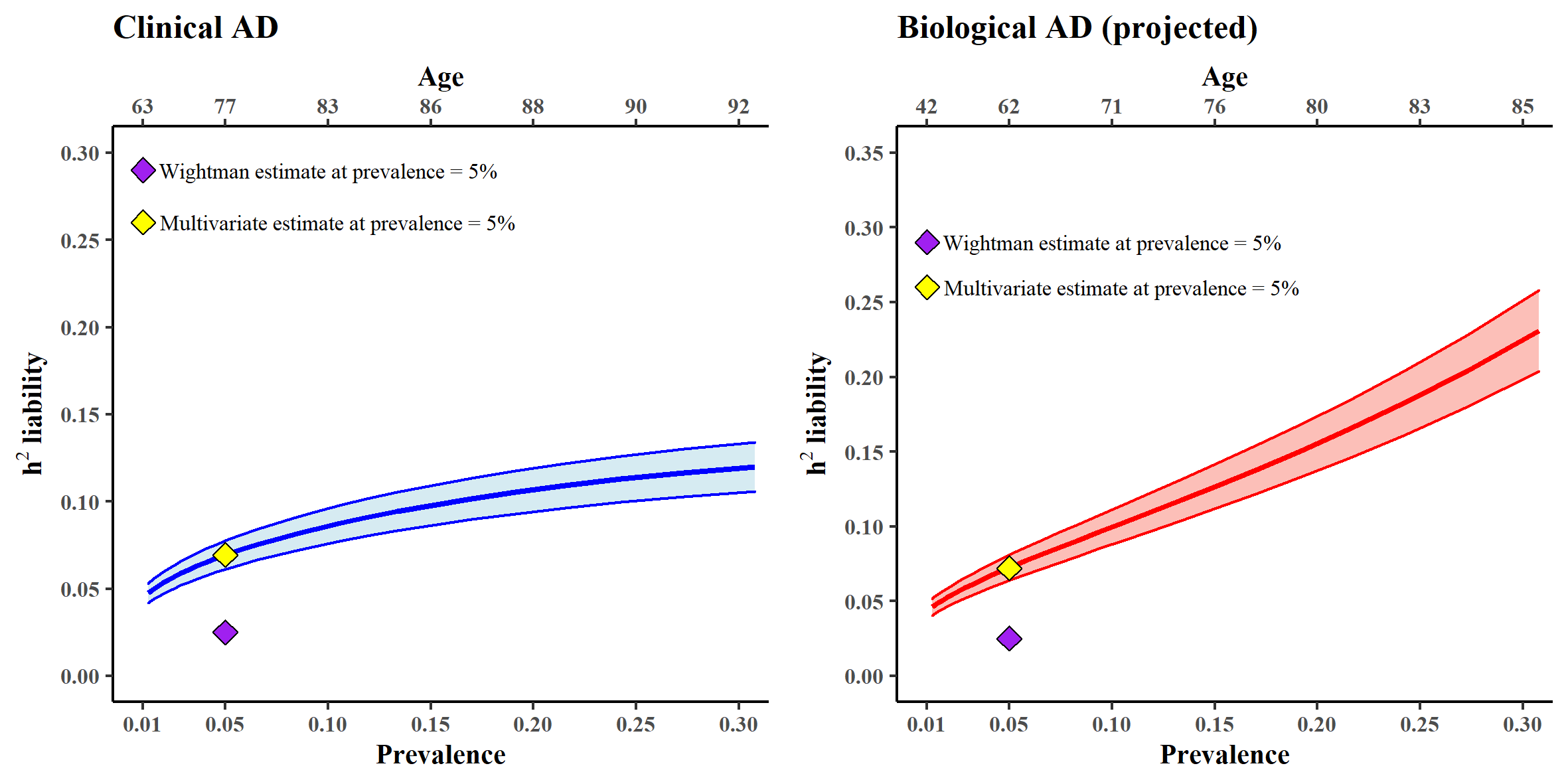

Supplement: S7 Fig — We provide rough approximations of the age equivalences of each prevalence rate on the top x axis. The purple diamond represents the estimate of 2.5% by Wightman et al.[4], which was based on an assumed population prevalence rate of 5%. The yellow diamond represents the estimate from the multivariate model introduced here, using the same assumed population prevalence rate of 5% (Clinical AD h2 = 0.069; Biological AD h2 = 0.072). The shading area around the line reflects +/- 1 SE of the h2 estimate. The steeper shift in the SNP heritability of AD for biological AD compared to clinical AD as a function of population prevalence stems from the correction for undetected biological AD within control participants who primarily only been screened for clinical AD. Thus, as the assumed prevalence rate of biological AD increases, the extent of case contamination in control participants increases, and the correction for undetected AD in control participants produces more dramatic increases in the projected heritability. (TIFF) [file pgen.1010208.s009.tiff]

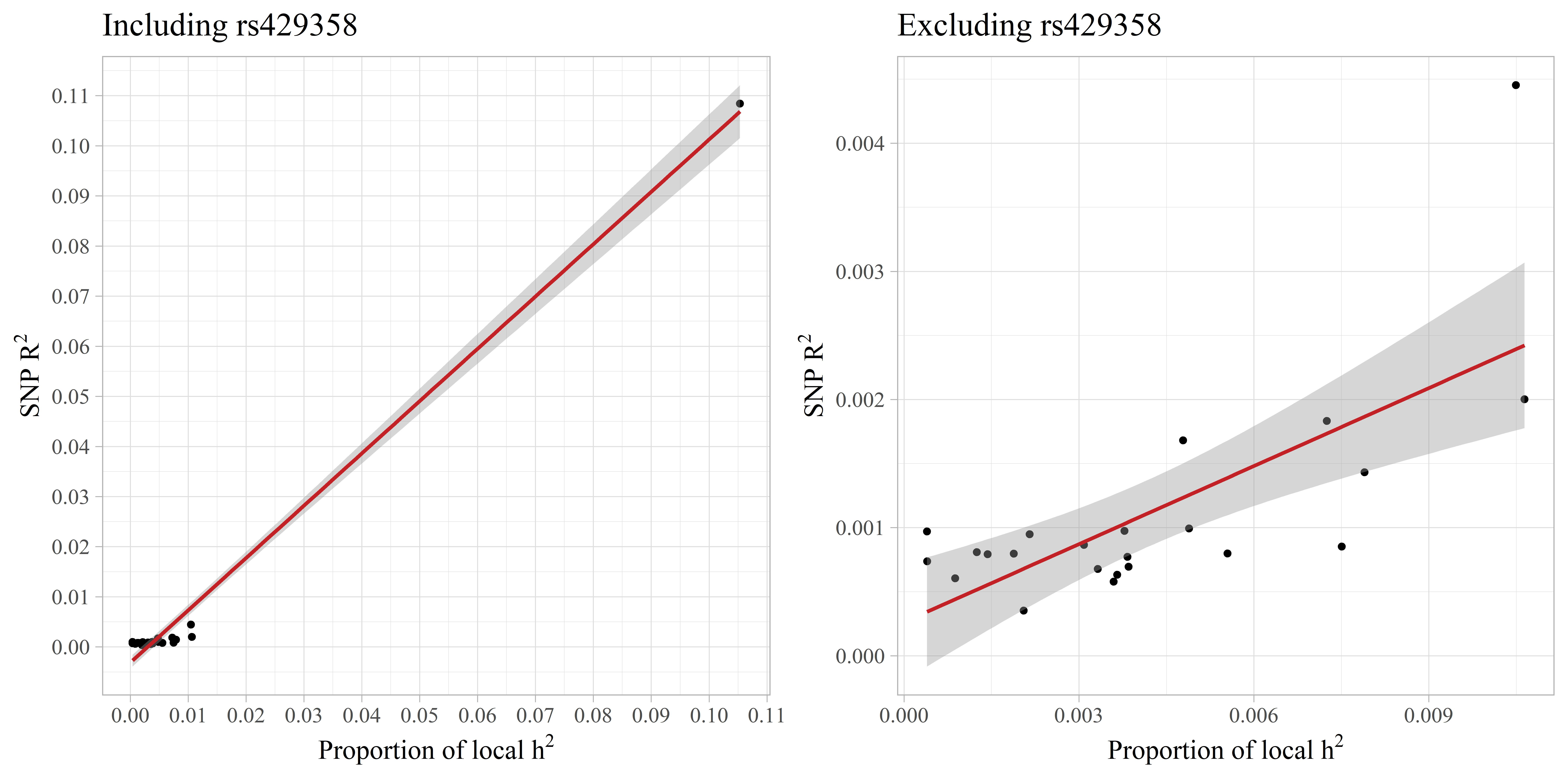

Supplement: S8 Fig — Association between local SNP heritability and GWAS effect sizes for GWAS loci that were genome wide significant in the multivariate GWAS including the APOE locus containing lead SNP rs429358 (left) and excluding this locus (right). (TIFF) [file pgen.1010208.s010.tiff]

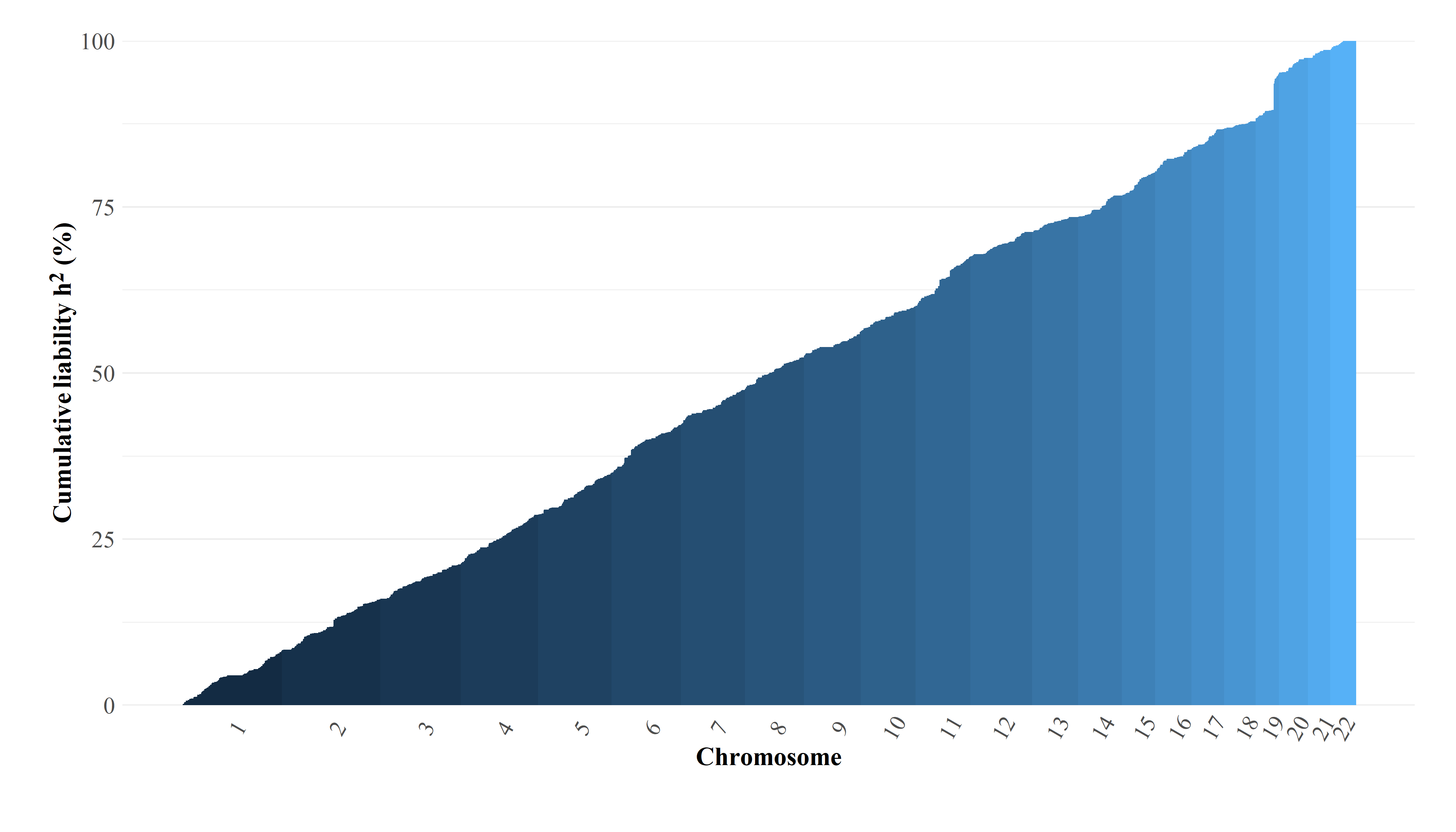

Supplement: S9 Fig — (TIFF) [file pgen.1010208.s011.tiff]

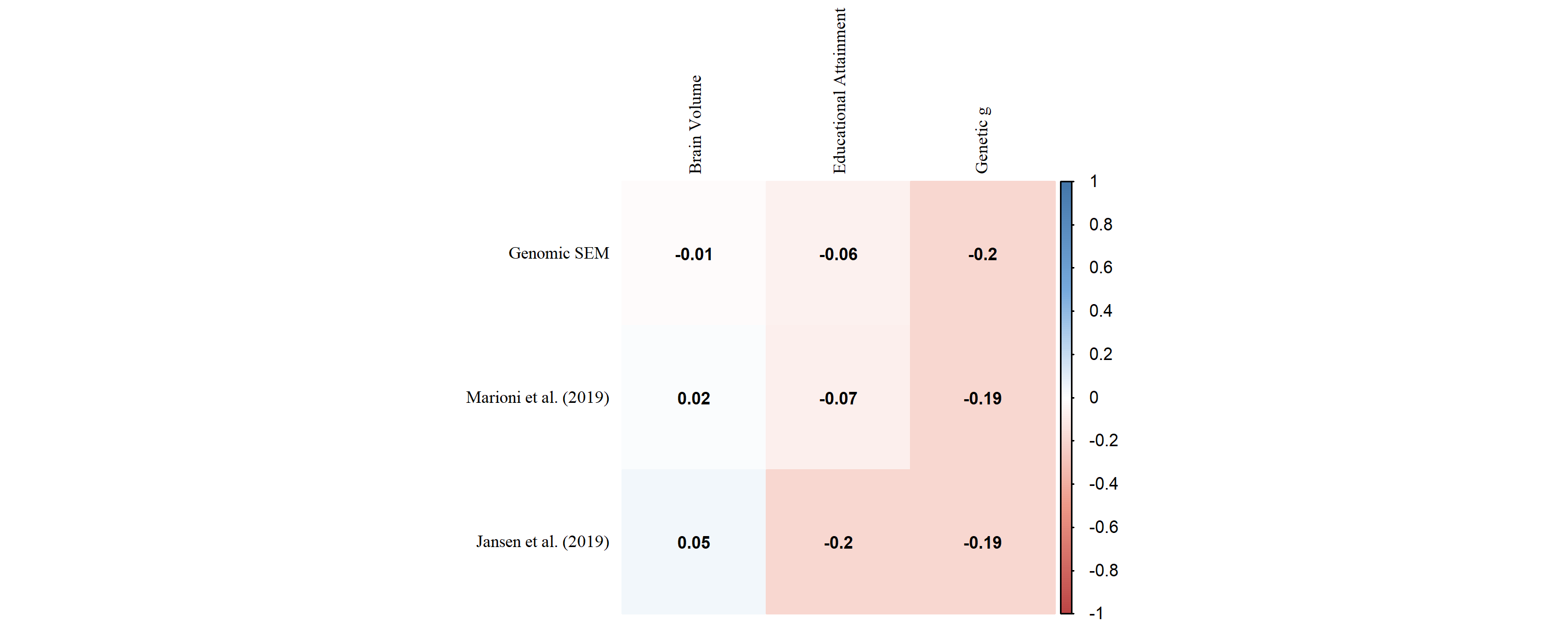

Supplement: S10 Fig — (TIFF) [file pgen.1010208.s012.tiff]
